# Single-Metal-Anchored 1D Mesoporous Channels to Enable Accelerated Redox Kinetics for Lithium-Sulfur Batteries

**DOI:** 10.1007/s40820-026-02177-w

**Published:** 2026-04-21

**Authors:** Dequn Zhao, Shun Wang, Yanan Zhang, Xingxing Zhang, Xuehan Hou, Hong Wang, Xiangyu Liu, Feiyang Yin, Wei Zhou, Wenhuan Huang

**Affiliations:** 1 Key Laboratory of Chemical Additives for China National Light Industry, College of Chemistry and Chemical Engineering, Shaanxi University of Science and Technology, Xi’an, 710021 People’s Republic of China; 2 Flexible Energy Storage and Interfacial Chemistry Key Laboratory of Shaanxi University, College of Chemistry and Chemical Engineering, Shaanxi University of Science and Technology, Xi’an, 710021 People’s Republic of China; 3 Xi’an Key Laboratory of Advanced Photo-Electronics Materials and Energy Conversion Device, Technological Institute of Materials & Energy Science (TIMES), Xijing University, Xi’an, 710123 People’s Republic of China; 4 Shanghai Lingfang Energy Co. Ltd., Shanghai, 201100 People’s Republic of China; 5 State Key Laboratory of High-Efficiency Utilization of Coal and Green Chemical Engineering, College of Chemistry and Chemical Engineering, Ningxia University, Yinchuan, 750021 People’s Republic of China; 6 School of Chemistry and Chemical Engineering, Hainan University, 58 Renmin Avenue, Haikou, 570228 People’s Republic of China

**Keywords:** Azolate hybrid frameworks, Lithium-sulfur batteries, Polysulfide conversion, Li^+^ flux, Dual-function regulation

## Abstract

Metal–organic frameworks (MOFs) have been demonstrated as promising separators for lithium-sulfur batteries (LSBs) owing to their highly tunable porous structures and intrinsic metal sites, which can guide uniform Li^+^ deposition, catalyze polysulfide conversion, and suppress polysulfide shuttling. However, conventional MOFs often have insufficient catalytic activity and Li^+^ transport control, and the role of their internal pore structure in regulating Li^+^ flux and polysulfide conversion remains unclear, limiting their effectiveness as high-performance separators. Herein, we report a series of azolate hybrid frameworks (M-AHF-DPDC, M = Fe, Co, Ni) featuring one-dimensional anionically charged channels that implement a dual-function regulation mechanism, simultaneously promoting uniform Li^+^ flux and catalyzing polysulfide conversion. Incorporation of Fe centers significantly enhances polysulfide redox kinetics, resulting in superior electrochemical performance, including a high initial capacity of 1400.7 mAh g^−1^ and stable cycling over 700 cycles at 1 C, along with uniform Li^+^ deposition, outperforming most reported MOF-based separators. Density functional theory calculations confirm that Fe sites strongly adsorb and catalytically convert diverse polysulfides, promoting rapid sulfur species transformation. This work demonstrates that the synergistic combination of polysulfide blocking and catalytic conversion enhances LSBs performance and offers a feasible strategy for high-energy–density rechargeable lithium-sulfur batteries.

**Supplementary Information:**

The online version contains supplementary material available at 10.1007/s40820-026-02177-w.

## Highlights

- An innovative azolate hybrid framework featuring dual-functional one-dimensional lithiophilic channels was constructed and applied to designing M-AHF-DPDC (M = Fe, Co, Ni) separator. Its unique framework design, equipped with lithiophilic channels and dense Fe active sites, enables uniform Li^+^ transport and efficient catalytic activity.
- The Fe-AHF-DPDC-modified separator significantly enhances the cycling stability and energy density of Li–S batteries while effectively suppressing the polysulfide shuttle effect.
- Through in situ Raman spectroscopy and density functional theory calculations, the strong adsorption capability and catalytic conversion mechanism of Fe-AHF-DPDC toward lithium polysulfides were elucidated.

**Supplementary Information:**

The online version contains supplementary material available at 10.1007/s40820-026-02177-w.

## Graphical abstract

**Figure:**
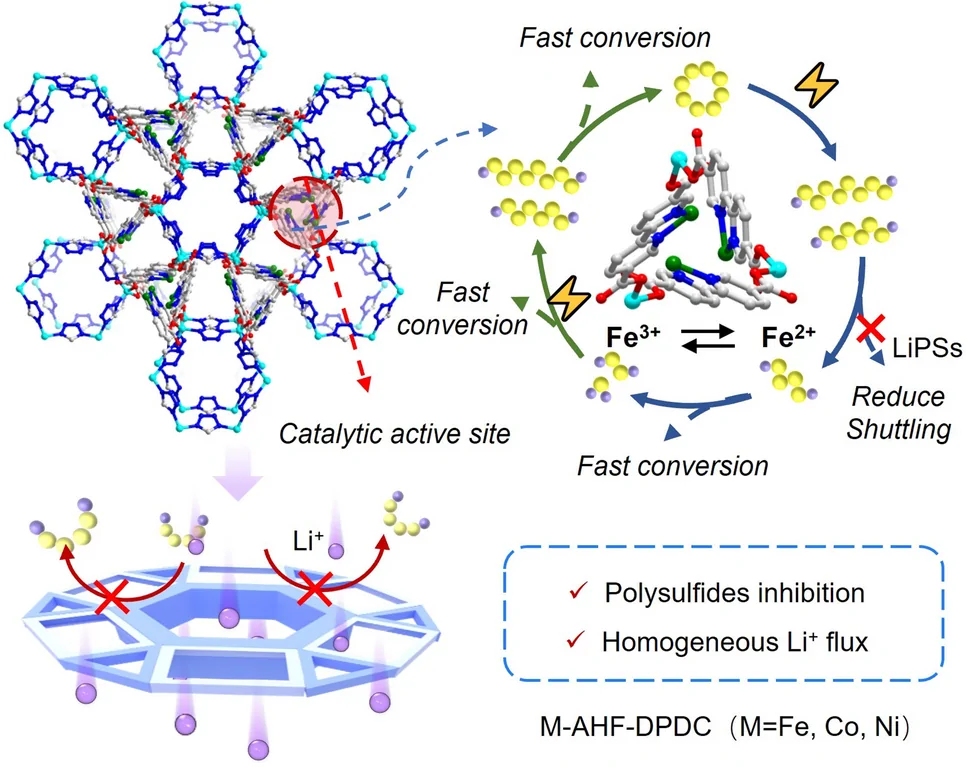

## Introduction

Lithium-sulfur batteries (LSBs) have become a major contender for next-generation energy storage systems due to their superior theoretical energy density of 2600 Wh kg^−1^ [1]. This advantage is further reinforced by the abundance of sulfur resources, environmental friendliness, and relatively low cost [2, 3]. Compared with traditional lithium-ion batteries, lithium-sulfur batteries have a higher energy density while offering a more sustainable outlook, making them advantageous in a wide range of applications from electric vehicles and aerospace technology to smart grid systems and portable electronic devices [4, 5]. However, despite these advantages, the commercialization of LSBs remains hindered by several critical challenges, among which the polysulfide (LiPSs) shuttle effect is the most prominent [6]. During charge/discharge processes, long-chain LiPSs (Li_2_S_n_, n = 4–8) dissolve in the electrolyte, migrate to the lithium anode, and undergo irreversible side reactions, leading to active material loss, rapid capacity fading, low Coulombic efficiency, uneven lithium deposition, and shortened cycle life [7–12]. Moreover, the accumulation of LiPSs disrupts Li^+^ transport, generating local concentration gradients that exacerbate dendrite formation and interfacial instability [13–15]. Therefore, regulating Li^+^ transport is crucial for stable and safe lithium deposition and stripping [16, 17].

To address these issues, various strategies have focused on designing advanced separator materials. Currently commercialized polyolefin separators (e.g., Celgard) possess good mechanical strength and thermal stability; however, they lack selective adsorption of LiPSs and cannot effectively control Li^+^ transport [18–20]. Metal–organic frameworks (MOFs), with tunable pore structures and intrinsic metal sites, have emerged as promising multifunctional separators [21–23]. MOFs can selectively block LiPSs migration via size-selective channels and incorporate metal centers as electrocatalytic sites to accelerate polysulfide conversion [24–26]. For example, Rameez Razaq et al. reported a Fe-ZIF-8-modified polypropylene (PP) separator, in which the highly ordered microporous structure of ZIF-8 allows Li^+^ passage while blocking LiPSs, and Fe-doped sites act as electrocatalysts [27, 28]. Recent studies further explored bimetallic alloy catalysts or biomimetic ion channel designs to enable bidirectional high-efficiency LiPSs conversion and controlled Li^+^ transport [29–31]. These advances indicate that rational design and optimization of MOF-based separators can overcome key bottlenecks in LSBs [32, 33].

Herein, we designed and constructed a one-dimensional nanoflower-like azolate hybrid framework, M-AHF-DPDC (M = Fe, Co, Ni) to implement a dual-transport regulation mechanism for Li^+^ and LiPSs. Its large 1D channels provide efficient pathways for rapid and uniform Li^+^ migration, stabilizing lithium deposition and suppressing dendrite formation. Simultaneously, Fe sites act as highly active electrocatalytic centers that adsorb and convert LiPSs, mitigating the shuttle effect. Incorporation of Fe significantly accelerates polysulfide redox kinetics, yielding excellent electrochemical performance: an initial capacity of 1400.7 mAh g^−1^ at 0.1 C, stable cycling over 700 cycles, 1157.35 mAh g^−1^ at 1 C and robust operation under high sulfur loading (4.89 mg cm^−2^) and lean electrolyte (E/S = 8 μL mg^−1^). Li||Li symmetric cells with Fe-AHF-DPDC separators demonstrate uniform Li^+^ deposition and stable cycling, surpassing most reported MOF-based separators. Further introduction of Zn^2+^ modulates the electronic structure of Fe, enhancing its catalytic activity and generating a synergistic effect. In situ Raman spectroscopy and density functional theory (DFT) calculations confirm strong adsorption and conversion of LiPSs by Fe-AHF-DPDC. This work highlights the critical role of Fe sites in 1D channels for promoting Li^+^ conduction and suppressing LiPSs transformation and shuttling, providing an effective design strategy for high-performance ion-conducting battery membranes.

## Experimental Section

### Preparation of Fe-DPDC, Co-DPDC and Ni-DPDC

1 mmol of 2, 2’-bipyridine-5, 5’-dicarboxylic acid, and 1 mmol of FeCl_2_·4H_2_O powder were weighed and added to a reactor containing 50 mL of deionized water, respectively. After stirring at 600 rpm for 3 days at room temperature, the mixture was filtered, washed three times with 20 mL of deionized water each time, and dried to obtain a light yellow powder designated as Fe-DPDC. For the preparation of pink Co-DPDC powder and green Ni-DPDC powder, all steps remained the same except for replacing FeCl_2_·4H_2_O with CoCl_2_·6H_2_O and NiCl_2_·6H_2_O powders.

### Preparation of AHF-DPDC, Fe-AHF-DPDC, Co-AHF-DPDC and Ni-AHF-DPDC

Briefly, 1 mmol of 2, 2’-bipyridine-5, 5’-dicarboxylic acid, and 1 mmol of 1H-tetrazole powder were weighed and added to a reactor containing 14 mL of N, N-dimethylacetamide (DMA). The solution was stirred at room temperature until homogeneous. Then, 1 mmol of zinc nitrate hexahydrate was added and stirred until completely dissolved. Subsequently, an appropriate amount of 4-methylammonium hydroxide solution was added to adjust the pH to a range of 6.7 ± 0.2. The reaction mixture was then heated to 120 °C for 2 days. After cooling to room temperature, the mixture was filtered and washed three times with 10 mL of DMAc each time. The AHF-DPDC crystals were obtained after drying. To prepare Fe-AHF-DPDC, Co-AHF-DPDC, or Ni-AHF-DPDC, all steps remained the same except for replacing DPDC with Fe-DPDC, Co-DPDC, or Ni-DPDC powder.

## Results and Discussion

### Design and Implantation of Active Sites in Lithium-Sulfur Batteries

In the initial phase, considering the singular catalytic mechanism of metal–organic frameworks, the introduction of metal catalytic centers proves essential for the conversion of polysulfides. We designed a porous anionic hybrid azolate frameworks (AHF) by incorporating Fe^2+^, Co^2+^ or Ni^2+^ metal catalytic centers into its structure. First, AHF-DPDC was synthesized based on tetrazole and 2,2′-bipyridine-5,5′-dicarboxylic acid (DPDC) ligands via a conventional solvothermal method, with detailed procedures provided in the Supporting Information. Subsequently, by employing a metallization strategy, Fe^2+^, Co^2+^ or Ni^2+^ ions were introduced onto the DPDC ligand, successfully synthesizing a series of ligand derivatives containing Fe^2+^, Co^2+^ or Ni^2+^ metal catalytic active centers, namely Fe-DPDC, Co-DPDC, and Ni-DPDC. Under solvothermal conditions, these ligands reacted with zinc acetate dihydrate as metal nodes and tetrazole, using tetrapropylammonium hydroxide as a structure-directing agent, ultimately yielding four isostructural MOF materials (AHF-DPDC and M-AHF-DPDC, M = Fe, Co, Ni) with high crystallinity, uniform particle size, and yields of 68%-72% (Figs. S1-S5).

**Fig. 1:**
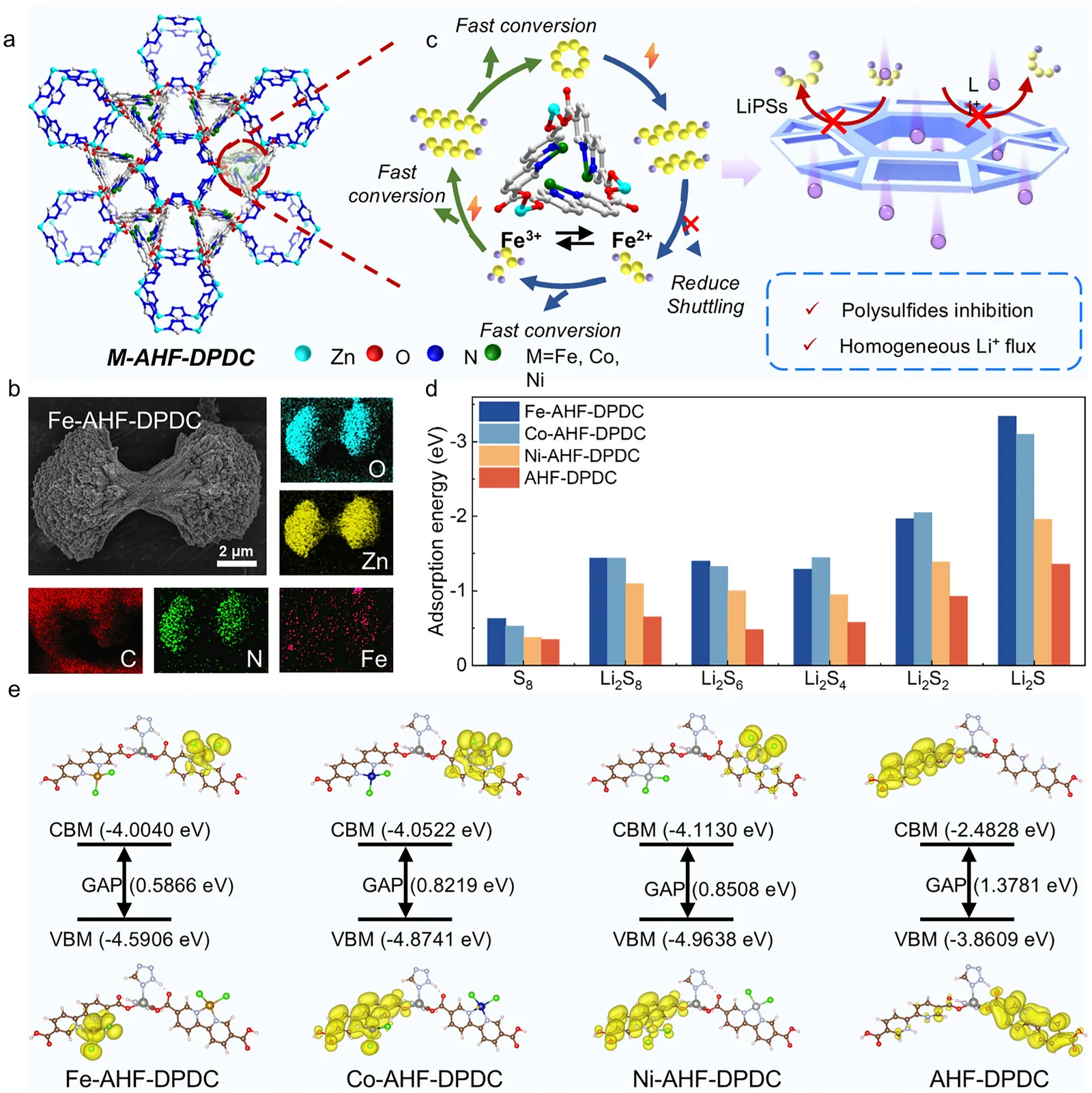
Materials structure characterization and polysulfide transformation kinetics. a, c Schematic illustration of Fe-AHF-DPDC for polysulfide conversion. **b** SEM images and corresponding elemental mapping of Fe-AHF-DPDC. **d** Calculated binding energy of Fe-AHF-DPDC, Co-AHF-DPDC, Ni-AHF-DPDC, and AHF-DPDC toward Li_2_S, Li_2_S_2_, Li_2_S_4_, Li_2_S_6_, Li_2_S_8_ and S_8_ by DFT. **e** Conduction band minimum (CBM) and valence band maximum (VBM) of Fe-AHF-DPDC, Co-AHF-DPDC, Ni-AHF-DPDC, and AHF-DPDC

We obtained highly crystalline microcrystalline powders, as evidenced by the structural simulation using the Zeo +  + software [34]. The topology of AHF-DPDC features six 4-connected Zn atoms serving as nodes coordinated by tetrazolate ligands to form 1D six-membered ring channels, with an aperture size of ~ 11.98 Å between adjacent rings and an interlayer spacing of ~ 37.89 Å. Parallel 1D tubes are further interconnected via linear DPDC linkers, constructing 1D triangular and hexagonal pore (Fig. 1a). And the hexagonal pore diameters of Fe-AHF-DPDC, Co-AHF-DPDC, Ni-AHF-DPDC, and AHF-DPDC are 15.3, 15.3, 14.9, and 17.1 Å (Fig. S6). The centrosymmetric arrangement of building units and the isotropic extension of the framework along the a and b axes result in the formation of 1D large channels along the c axis, giving rise to a complex 3D network structure. The unit cell parameters of AHF-DPDC are a = b = 32.3705 Å, c = 37.889 Å, α = β = 90°, γ = 120°, indicating that the MOF belongs to the space group of R-3(148)-trigonal, exhibiting high symmetry and periodic ordering.

Comparing the powder X-ray diffraction (PXRD) patterns of Fe-AHF-DPDC, Co-AHF-DPDC, Ni-AHF-DPDC, and AHF-DPDC demonstrates that all four materials have been successfully synthesized and exhibit high crystallinity. As shown in Fig. S7, the PXRD patterns of all samples display sharp and clear diffraction peaks that match well with the simulated patterns, indicating that Fe-AHF-DPDC, Co-AHF-DPDC, Ni-AHF-DPDC, and AHF-DPDC possess high crystallinity, structural integrity, and phase purity.

The morphologies of Fe-AHF-DPDC, Co-AHF-DPDC, Ni-AHF-DPDC, and AHF-DPDC were characterized by scanning electron microscopy (SEM), and it can be seen that all four crystals exhibit a dumbbell-shaped structure. EDS elemental mapping (Figs. 1b and S8-S10) indicates the presence of C, N, O, Zn, and Fe in Fe-AHF-DPDC; C, N, O, Zn, and Co in Co-AHF-DPDC; C, N, O, Zn, and Ni in Ni-AHF-DPDC, while only C, N, O, and Zn are present in AHF-DPDC. It can be observed that Fe, Co, and Ni are evenly distributed in their respective materials. Meanwhile, X-ray photoelectron spectroscopy (XPS) (Figs. S11-S14) analysis results indicate that during the synthesis of Fe-AHF-DPDC and Co-AHF-DPDC, Fe^2+^ and Co^2+^ are oxidized to Fe^3+^ and Co^3+^, respectively. In contrast, due to the thermodynamic instability of Ni^3+^ in this system, nickel in Ni-AHF-DPDC exists predominantly as Ni^2+^. These results demonstrate the successful chelation of these metal ions with the organic ligands.

To verify the successful formation of stable coordination bonds between the DPDC ligand and metal ions (including Zn^2+^ and other central metal ions) within the MOF structure, as well as the successful synthesis of the target materials, the Fourier transform infrared (FT-IR) spectroscopy results (Fig. S15) reveal that the characteristic absorption peaks observed in the 400–800 cm^−1^ range for Fe-AHF-DPDC, Co-AHF-DPDC, and Ni-AHF-DPDC can be attributed to the vibrational modes of pyridine rings, confirming the successful coordination between metal ions and pyridinic nitrogen atoms. Compared with the free DPDC ligand, the complete disappearance of hydroxyl (-OH) stretching vibration peaks in the 2500–3000 cm^−1^ range for all three MOF materials indicates the coordination interaction between Zn^2+^ ions and hydroxyl groups in the ligand. Significantly, the characteristic peak of carboxyl C = O stretching vibration at 1689 cm^−1^ undergoes a redshift to 1669 cm^−1^ in the MOF materials. This frequency reduction phenomenon originates from the enhanced sp^2^ hybridization and increased conjugation effect of carboxyl groups resulting from Zn^2+^-oxygen coordination, thereby providing further verification for the formation of Zn–O coordination bonds [35].

To investigate whether the Zn^2+^ sites in the bimetallic metal–organic framework can be replaced by other metal ions (M^2+^) through metal exchange, we designed a metal substitution experiment. We replaced DPDC with 4, 4′-biphenyldicarboxylic acid (BPDC) as the ligand, keeping the other synthesis steps the same as those for the synthesis of AHF-DPDC crystals. The resulting crystals, isostructural with AHF-DPDC (AHF-BPDC), were immersed in N, N-dimethylacetamide containing CoCl_2_·6H_2_O for 6 h. Subsequently, they were washed three times with 10 mL of DMA to remove surface-adsorbed metal ions. The Zn and Co contents before and after the treatment were determined by inductively coupled plasma (ICP) analysis (Table S1). The results showed that the mass percentage of Zn was 16.2% before treatment and 16.4% after treatment, indicating no significant change. At the same time, only trace amounts of Co (0.11%) were detected. This amount is close to the detection limit of the instrument and can be attributed to physical adsorption or residual impurities, rather than chemical substitution of the Zn^2+^ sites within the framework. This result indicates that under the mild conditions used, the coordination environment of Zn^2+^ in such metal–organic frameworks is very stable and metal ion exchange is unlikely to occur. Thermogravimetric analysis (TGA) (Fig. S16) of these crystals indicated that this class of crystals exhibits good thermal stability. These results further validate the effectiveness of the synthetic protocol and the structural fidelity of the resulting materials.

Notably, the framework features one-dimensional large channels alongside uniformly distributed triangular and hexagonal pores. The one-dimensional macroporous framework constructed from tetrazole and Zn^2+^ exhibits an anionic backbone that effectively guides uniform Li^+^ deposition, thereby significantly suppressing lithium dendrite growth. Compared to AHF-DPDC, Fe/Co/Ni-AHF-DPDC crystals incorporate abundant transition metal-modified active sites. We propose that these metal centers serve as highly efficient catalytic sites, capable of adsorbing and activating LiPS intermediates, substantially lowering the activation energy of their conversion reactions and thus dramatically enhancing the reaction kinetics of LiPSs in LSBs. This synergistic effect also effectively mitigates the “shuttle effect” (Fig. 1c).

To gain theoretical insight into the interfacial interactions, DFT calculations were performed to compare the relative binding strengths between various sulfur species and the MOF fragment. While acknowledging the complexity of practical interfaces, these standardized calculations provide a consistent basis for evaluating comparative trends, which are crucial for understanding the role of interfacial binding in catalytic performance (Figs. S17-S21). As shown in Fig. 1d, the calculated binding energies of LiPS intermediates reveal a clear trend: Fe-AHF-DPDC exhibits substantially stronger binding for S_8_ (0.63 eV), Li_2_S_8_ (1.44 eV), Li_2_S_6_ (1.40 eV), and Li_2_S (3.34 eV) compared to Co-AHF-DPDC, Ni-AHF-DPDC, and AHF-DPDC. This consistent relative ordering suggests that the Fe sites provide superior binding and confinement capability for diverse sulfur species compared to other metal centers, thereby likely enhancing the anchoring and catalytic conversion of polysulfides.

To clarify the effect of incorporating transition metal centers on the electronic structure and their catalytic conversion ability toward polysulfides, DFT calculations were performed to evaluate the band gaps of Fe-AHF-DPDC, Co-AHF-DPDC, Ni-AHF-DPDC, and AHF-DPDC (i.e., the energy difference between the conduction band minimum and the valence band maximum), as shown in Fig. 1e. Fe-AHF-DPDC has the smallest band gap (0.5866 eV), compared with 0.8219 eV for Co-AHF-DPDC, 0.8508 eV for Ni-AHF-DPDC, and 1.3781 eV for AHF-DPDC, indicating that electrons in Fe-AHF-DPDC can more easily transition from the valence band to the conduction band. This enhanced electronic conductivity promotes stronger chemical interactions between the Fe sites and LiPSs, thereby facilitating efficient adsorption and catalytic conversion of polysulfides. As a result, this helps suppress the shuttle effect and improves the cycling stability and electrochemical performance of lithium-sulfur batteries.

According to the calculation results, these four MOF materials were applied to modify separators in lithium-sulfur batteries. Fe-AHF-DPDC, Co-AHF-DPDC, Ni-AHF-DPDC, and AHF-DPDC powders were mixed with conductive carbon black (super-P Li) and polyvinylidene fluoride (PVDF) in a mass ratio of 7:2:1, respectively. After thorough grinding, an appropriate amount of N-methyl-2-pyrrolidone (NMP) was added and stirred to form a uniform slurry. Then, using a doctor blade coating method, the slurry was applied onto pretreated Celgard™ 2500 separator substrates (thickness: 25 ± 1 μm), forming modified separators with a thickness of approximately 50 ± 5 μm (Fig. S22). Optical images of the modified separators (Fig. S23) show that the MOF layer fully covers the PP substrate. As shown in Fig. S24, the Fe-AHF-DPDC-modified membrane maintains its original shape even after being twisted twice, indicating its strong adhesion and excellent mechanical flexibility. The mechanical and thermal stability of the membrane is crucial for the battery to withstand extreme operating conditions, helping the electrodes maintain close contact and reducing interfacial resistance.

The thermal stability test carried out under ambient air conditions is shown in Fig. S25. Compared with PP, the incorporation of Fe-AHF-DPDC, Co-AHF-DPDC, Ni-AHF-DPDC, or AHF-DPDC into the membranes significantly improved their heat resistance [36]. When the temperature was increased from 25 to 150 °C, the PP membrane completely melted, while the AHF-DPDC-modified membrane only underwent partial deformation. In contrast, the Fe-AHF-DPDC, Co-AHF-DPDC, and Ni-AHF-DPDC-modified membranes showed almost no dimensional changes, demonstrating excellent thermal stability. These results indicate that the MOF structures constructed from metal node assemblies significantly enhance the thermal resistance of the membranes, suggesting that bimetallic MOF-modified membranes can maintain their shape without melting, shrinking, or deforming during battery charging and discharging or overheating under high-temperature or extreme operating conditions, thereby improving safety and cycle life.

In order to test the ability of the membrane surface to be wetted by the electrolyte and its hydrophobicity, thereby evaluating whether lithium ions can quickly and evenly pass through the membrane pores, enhancing ionic conductivity and reducing interfacial resistance, contact angle (CA) measurements were used to assess the wettability of different membranes with deionized water (Fig. S26) and electrolyte (Fig. S27) [37, 38]. The results show that the water contact angle of the Fe-AHF-DPDC-modified separator is 146.6°, higher than that of Co-AHF-DPDC (143.4°), Ni-AHF-DPDC (143.1°), AHF-DPDC (127.3°), and PP (118°). Notably, the electrolyte contact angle of the Fe-AHF-DPDC separator is only 6.37°, much lower than that of Co-AHF-DPDC (7.9°), Ni-AHF-DPDC (8.9°), AHF-DPDC (11.7°), and pristine PP (47.6°). This significant reduction in the electrolyte contact angle indicates that the Fe-AHF-DPDC-modified separator has excellent surface wettability, which helps to efficiently absorb the electrolyte and facilitate rapid lithium migration at the interface.

### Active Site Fe Promotes the Catalytic Redox Reactions of LiPSs

The membrane plays a crucial role in promoting the transport of lithium ions between the anode and cathode in LSBs [39]. Therefore, ion conductivity tests were conducted on four modified membranes, as shown in Fig. S28. Fe-AHF-DPDC exhibited the highest lithium-ion conductivity (σ = 1.98 × 10^–3^ S cm^−1^), surpassing Co-AHF-DPDC (1.05 × 10^–3^ S cm^−1^), Ni-AHF-DPDC (9.25 × 10^–4^ S cm^−1^), and AHF-DPDC (5.03 × 10^–4^ S cm^−1^). Additionally, the lithium-ion transference numbers (*t*_*Li*_^+^) obtained from direct current (DC) polarization tests (Fig. S29) were 0.6047, 0.5837, 0.5455, and 0.4506 for Fe-AHF-DPDC, Co-AHF-DPDC, Ni-AHF-DPDC, and AHF-DPDC, respectively. These results indicate that Fe-AHF-DPDC can achieve faster charge–discharge rates and superior lithium-ion transport kinetics.

**Fig. 2:**
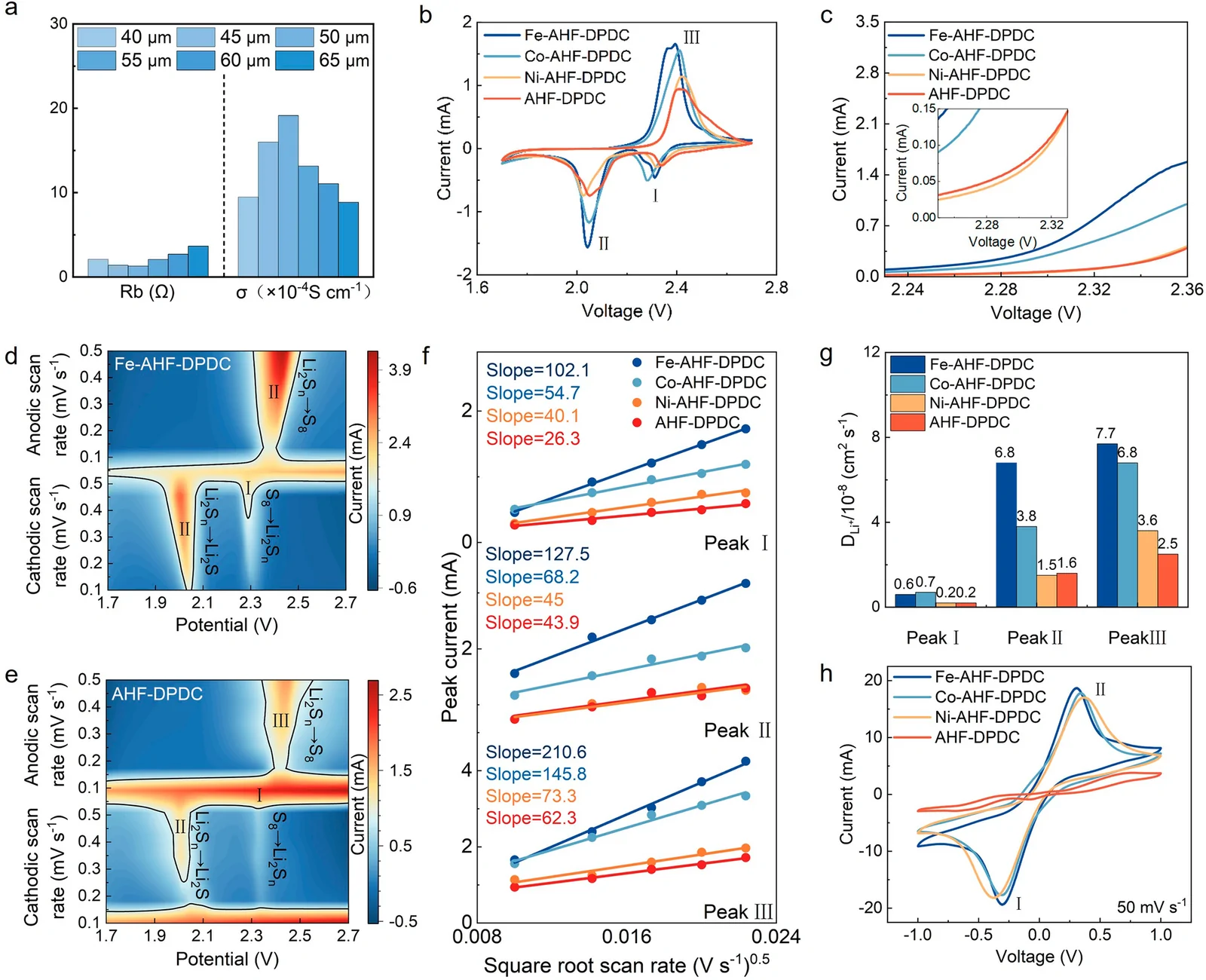
Kinetic catalytic process of polysulfides. **a** Ionic conductivity of Fe-AHF-DPDC at different thicknesses. **b** CV curves of Fe-AHF-DPDC, Co-AHF-DPDC, Ni-AHF-DPDC and AHF-DPDC batteries at a scan rate of 0.1 mV s^−1^. **c** LSV curves at Peak III for Fe-AHF-DPDC, Co-AHF-DPDC, Ni-AHF-DPDC and AHF-DPDC batteries. (The inset shows a magnified view of the highlighted region.) **d** Contour plots of CV curves for the Fe-AHF-DPDC battery at various scan rates. **e** Contour plots of CV curves for the AHF-DPDC battery at various scan rates. **f** Linear fitting of peak currents for Fe-AHF-DPDC, Co-AHF-DPDC, Ni-AHF-DPDC and AHF-DPDC batteries. **g** Ion diffusion coefficients. **h** Cyclic voltammetry (CV) curves of symmetric cells based on Fe-AHF-DPDC, Co-AHF-DPDC, Ni-AHF-DPDC and AHF-DPDC

In order to systematically study the effect of different thicknesses of Fe-AHF-DPDC-modified membranes on ionic conductivity, a series of Fe-AHF-DPDC membranes with thicknesses of 40, 45, 50, 55, 60, and 65 μm were prepared using the doctor blade coating method (Fig. S30). 25 μL of electrolyte was added to each side of the membrane, and they were assembled into stainless steel symmetric cells to perform EIS tests to evaluate ionic conduction performance. The results indicate that an overly thick separator increases the migration distance and resistance of Li^+^, thereby reducing ionic conductivity. Conversely, a separator that is too thin may lack sufficient active material to effectively prevent LiPSs from diffusing from the cathode to the anode. These side reactions could form an insulating layer on the electrode surface, further hindering Li^+^ transport. For Fe-AHF-DPDC, the optimal thickness is 50 μm, which achieves a maximum conductivity of 1.98 × 10^–3^ S cm^−1^; therefore, this thickness was used in all subsequent experiments (Figs. 2a and S31). The electrochemical performance of M-AHF-DPDC and AHF-DPDC separators was evaluated in CR-2032 coin cells with the ~ 1.0 mg cm^−2^ (content of 75%) S@CNT cathode. Cyclic voltammetry (CV) measurements were employed to study the redox kinetics of LiPSs species on the functionalized separators [29]. As shown in Fig. 2b, at a scan rate of 0.1 mV s^−1^, the cyclic voltammetry curves of LSBs with different separators display two reduction peaks at 2.312 V (Peak I) and 2.04 V (Peak II), corresponding to the conversion of Li_2_S_8_ to Li_2_S_6_/Li_2_S_4_ and its further reduction to Li_2_S, respectively. Meanwhile, the oxidation peak at 2.364 V (Peak III) corresponds to the oxidation of Li_2_S_2_/Li_2_S back to long-chain polysulfides and finally to solid-state S_8_. Compared with Co-AHF-DPDC, Ni-AHF-DPDC, and AHF-DPDC, the Fe-AHF-DPDC cell exhibits the smallest voltage hysteresis, the largest CV area, and the highest peak current intensity, indicating more efficient catalytic conversion of LiPSs, higher sulfur utilization, and significantly accelerated redox kinetics [25, 40].

To further evaluate the catalytic activity during the sulfur conversion process, linear sweep voltammetry (LSV) experiments were conducted using Fe-AHF-DPDC, Co-AHF-DPDC, Ni-AHF-DPDC, and AHF-DPDC-modified separators to assess the oxidation kinetics of Li_2_S (Figs. 2c and S32). The Tafel slopes obtained from Peak II and Peak III (Figs. S33 and S34) serve as key indicators of electrocatalytic efficiency, where smaller values indicate higher catalytic activity. For the cathodic peak II (around 2.36 V) and the anodic peak III (around 2.04 V), Fe-AHF-DPDC exhibits the lowest Tafel slopes, at 85.1 and 72.7 mV dec^−1^, respectively, while Co-AHF-DPDC has Tafel slopes of 115.8 and 110.3 mV dec^−1^, Ni-AHF-DPDC has 170.2 and 127.2 mV dec^−1^, and AHF-DPDC has 490.2 and 162.5 mV dec^−1^. These results demonstrate that Fe-AHF-DPDC has stronger catalytic activity toward the LiPSs redox reactions, which helps to improve the performance of LSBs [41].

Cyclic voltammetry (CV) measurements were performed at scan rates ranging from 0.1 to 0.5 mV s^−1^ to further analyze the reaction kinetics and lithium diffusion capabilities of the four separators. As shown in Figs. 2d, 2e, S35, and S36, contour plots of CV curves for Fe-AHF-DPDC, Co-AHF-DPDC, Ni-AHF-DPDC, and AHF-DPDC batteries are presented. As the scan rate increases, the polarization between the cathodic peaks (Peak I and Peak II) and anodic peak (Peak III) becomes more pronounced, indicating that the reduction of sulfur and the lithium insertion/extraction processes must be completed in a shorter time. In diffusion-controlled processes, when the potential changes rapidly, reactants may not reach the electrode surface in time, or the products may not diffuse effectively, leading to an increase in overpotential [19].

The linear relationship between the redox peak current *I*_*p*_) of the four membranes and the square root of the scan rate (*ν*^0.5^) is plotted in Figs. 2f and S37, which is consistent with a typical diffusion-limited process. The slope of *I*_*p*_ vs. *ν*^0.5^ for Fe-AHF-DPDC is steeper than those for Co-AHF-DPDC, Ni-AHF-DPDC, and AHF-DPDC, indicating faster lithium diffusion during the redox reaction. CV tests conducted at scan rates ranging from 0.1 to 0.5 mV s^−1^ were used to further calculate the lithium diffusion coefficient (*D*_*Li*_^+^) (Fig. 2g). According to the classical Randles–Ševčík equation [42]:

1 $$ Ip = \left( {2.69 \times 10^{5} } \right)n^{1.5} AD_{Li}^{ + 0.5} C_{Li}^{ + } v^{0.5} $$

where *Ip* is the peak current, *n* is the number of electrons transferred (*n* = 2), *A* is the electrode area (0.785 cm^2^), *C*_*Li*_^+^ is the Li^+^ concentration, and v is the scan rate. Thus, the slope of the *Ip*-*v*^0.5^ plot is directly proportional to *D*_*Li*_^+^. Based on the LSV-derived peaks (I, II, and III), the calculated *D*_*Li*_^+^ values for Fe-AHF-DPDC, Co-AHF-DPDC, Ni-AHF-DPDC, and AHF-DPDC are 5.85 × 10^–9^, 7.15 × 10^–9^, 2.33 × 10^–9^, and 1.98 × 10^–9^ cm^2^ s^−1^; 6.82 × 10^–8^, 3.84 × 10^–8^, 1.54 × 10^–8^, and 1.55 × 10^–8^ cm^2^ s^−1^; and 7.72 × 10^–8^, 6.82 × 10^–8^, 3.64 × 10^–8^, and 2.50 × 10^–8^ cm^2^ s^−1^, respectively. Fe-AHF-DPDC demonstrates the highest Li^+^ diffusivity, highlighting its ability to accelerate sulfur conversion and enhance LiPSs redox kinetics, thereby suppressing the shuttle effect and boosting MOF shell catalytic activity. These results collectively indicate that the sulfur redox reaction (SRR) is strongly influenced by the Fe-AHF-DPDC center, revealing synergistic catalytic effects [43].

To further investigate the redox reaction kinetics of the four modified separators, CV tests were conducted on symmetric cells containing 5 mM Li_2_S_6_ in the electrolyte at a scan rate of 50 mV s^−1^ [44]. As shown in Fig. 2h, the symmetric cell based on Fe-AHF-DPDC exhibits distinct redox peaks at -0.31 V (reduction) and 0.31 V (oxidation), corresponding to the reduction of Li_2_S_6_ to lower-order lithium polysulfides (Li_2_S_2_/Li_2_S) and the oxidation of Li_2_S back to S_8_, respectively. From Fig. S38, the overpotentials at ± 0.31 V for Fe-AHF-DPDC, Co-AHF-DPDC, Ni-AHF-DPDC, and AHF-DPDC are 0.617, 0.659, 0.752, and 1.055 V, respectively. It is noteworthy that Fe-AHF-DPDC exhibits a highly reversible redox pair with the lowest overpotential and the highest current response, indicating that Fe-doped MOFs possess strong catalytic activity in accelerating the redox kinetics of LiPSs adsorption.

To evaluate the ability of Fe-AHF-DPDC, Co-AHF-DPDC, Ni-AHF-DPDC, and AHF-DPDC-modified separators to suppress LiPSs shuttling, H-type glass tube tests were conducted (Fig. S39). In this setup, the left chamber contained 0.25 M Li_2_S_6_ dissolved in DOL/DME (1:1 v/v), while the right chamber contained pure DOL/DME electrolyte. Driven by the concentration gradient, after standing for 12 h, the right chamber with Fe-AHF-DPDC remained clear, indicating slow LiPSs diffusion. In contrast, Co-AHF-DPDC, Ni-AHF-DPDC, and AHF-DPDC showed noticeable LiPSs migration. Additionally, LiPSs diffused fastest through the PP separator, confirming the superiority of Fe-AHF-DPDC in suppressing the diffusion of LiPSs into ether-based electrolytes [45].

**Fig. 3:**
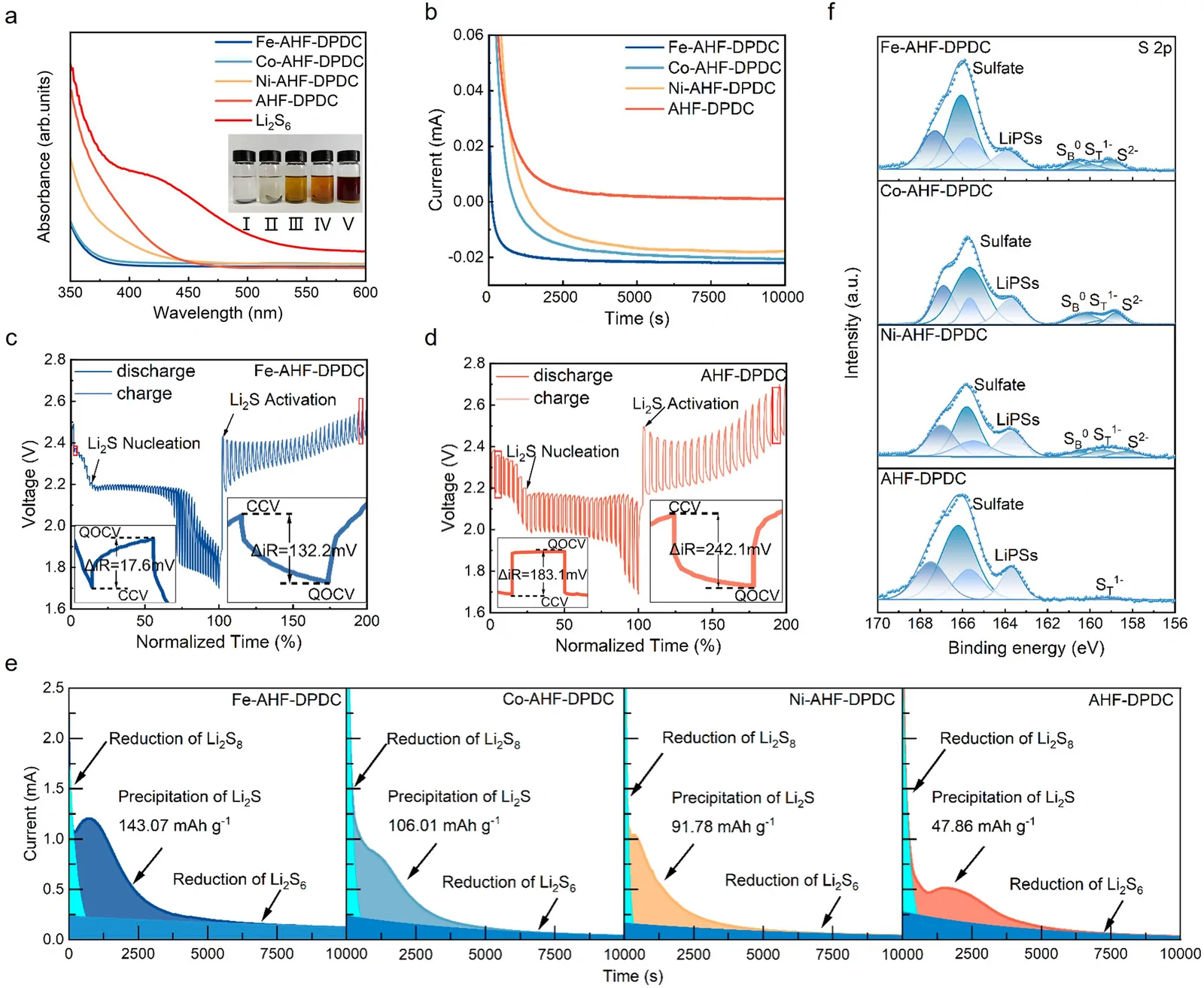
Insights into the static sulfur conversion in LSBs using Fe-AHF-DPDC, Co-AHF-DPDC, Ni-AHF-DPDC and AHF-DPDC separators. **a** UV–Vis absorption spectra of Li_2_S_6_ solutions after adsorption by Fe-AHF-DPDC, Co-AHF-DPDC, Ni-AHF-DPDC and Zn-AHF-DPDC separators. (I: Fe-AHF-DPDC; II: Co-AHF-DPDC; III: Ni-AHF-DPDC; IV: AHF-DPDC; V: Li_2_S_6_.) **b** Shuttle current at 2.38 V. **c** GITT curves of the Fe-AHF-DPDC battery during the first discharge/charge process at a current density of 0.1 C. **d** GITT curves of the AHF-DPDC battery during the first discharge/charge process at a current density of 0.1 C. **e** Typical current–time profile of Li_2_S deposition for Fe-AHF-DPDC, Co-AHF-DPDC, Ni-AHF-DPDC, and Zn-AHF-DPDC at ~ 2.06 V. **f** S 2*p* XPS spectra of the separators after 50 cycles at 1 C for Fe-AHF-DPDC, Co-AHF-DPDC, Ni-AHF-DPDC, and AHF-DPDC batteries

To compare the adsorption abilities of Fe-AHF-DPDC, Co-AHF-DPDC, Ni-AHF-DPDC, and AHF-DPDC toward LiPSs, as shown in Fig. S40, these four MOF-modified membranes were immersed in a Li_2_S_6_ solution. After standing for 24 h, the solution containing Fe-AHF-DPDC became almost colorless, while Co-AHF-DPDC appeared light yellow, and Ni-AHF-DPDC and AHF-DPDC remained yellow. The original solution without any MOF remained deep yellow. In addition, the UV–visible spectrum of the Li_2_S_6_ solution treated with Fe-AHF-DPDC showed a significant decrease in absorption peaks (Fig. 3a), further confirming that Fe-AHF-DPDC has a strong adsorption ability for LiPSs [46].

To demonstrate the effectiveness of Fe-AHF-DPDC in inhibiting the LiPSs shuttle effect, shuttle current measurements were conducted at 2.38 V after three battery cycles (Fig. 3b). Compared to Co-AHF-DPDC, Ni-AHF-DPDC, and AHF-DPDC batteries, the Fe-AHF-DPDC battery showed significantly lower shuttle currents. This result indicates that Fe-AHF-DPDC can effectively suppress the diffusion of LiPSs, thereby achieving higher sulfur utilization and reduced capacity decay, strongly inhibiting polysulfides, promoting uniform lithium deposition, and suppressing dendrite formation.

The impact of modified separators on the sulfur conversion kinetics in LSBs was investigated via galvanostatic intermittent titration technique (GITT). As shown in Figs. 3c, d and S41, the Fe-AHF-DPDC-modified battery exhibited smaller IR drops during both quasi-open-circuit voltage (QOCV, 17.6 mV) and closed-circuit voltage (CCV, 132.2 mV) phases compared to those with Co-AHF-DPDC, Ni-AHF-DPDC, and AHF-DPDC separators. This indicates that Fe-AHF-DPDC effectively reduces the energy barrier for polysulfide conversion, promoting oxygen-assisted sulfur redox kinetics [5].

Furthermore, the dissolution process of S_8_ (Fig. S42) accounted for approximately 18.43% (9.42/51.1 h), 25.05% (10.92/43.6 h), 25.08% (7.42/29.59 h), and 28.47% (9.42/33.09 h) of the discharge stage for Fe-AHF-DPDC, Co-AHF-DPDC, Ni-AHF-DPDC, and AHF-DPDC, respectively. The relatively smaller fraction in the modified separators can be attributed to more efficient Li_2_S nucleation [2].

The reduction and precipitation behavior of Li_2_S was studied through potentiostatic discharge to evaluate the electrocatalytic performance of Fe-AHF-DPDC, Co-AHF-DPDC, Ni-AHF-DPDC, and AHF-DPDC for short-chain Li_2_S. The current–time (i–t) curves are shown in Fig. 3e. First, the cell was discharged at 2.10 V to completely consume the long-chain Li_2_S_n_ (n = 4–8), followed by polarization at 2.05 V to induce Li_2_S nucleation/growth until the current dropped below 10^–5^ A. The Fe-AHF-DPDC separator achieved a Li_2_S deposition capacity of 143.07 mAh g^−1^, significantly higher than those of Co-AHF-DPDC (106.01 mAh g^−1^), Ni-AHF-DPDC (91.78 mAh g^−1^), and AHF-DPDC (47.86 mAh g^−1^). SEM images of Li_2_S deposited on the surfaces of different separators (Fig. S43) reveal that Li_2_S formed uniformly and densely on Fe-AHF-DPDC, whereas the deposition was non-uniform and sparse on Co-AHF-DPDC, Ni-AHF-DPDC, and AHF-DPDC. These results demonstrate that Fe-AHF-DPDC promotes uniform Li_2_S deposition [47–49], accelerates its precipitation kinetics, enhances sulfur utilization, and improves catalytic conversion in LSBs.

To verify the catalytic effect of the modified separators, LSBs were assembled using Fe-AHF-DPDC, Co-AHF-DPDC, Ni-AHF-DPDC, and AHF-DPDC separators and cycled for 50 cycles at 1 C. Then, X-ray photoelectron spectroscopy (XPS) (Fig. 3f) was used to analyze the phase composition on the surface of each separator. During the cycling process, sulfur in the cathode underwent a reduction process, transforming from S_8_ molecules to LiPSs (Li_2_S_n_), eventually forming the discharge products Li_2_S_2_ and Li_2_S. The gradual reduction of these sulfur species leads to an increase in the electron density around the sulfur atoms. Therefore, the overall trend of sulfur chemical state evolution during the cycle manifests as an increase in electron cloud density, which is reflected in the XPS spectra as a shift of the binding energy toward lower energies. Through the analysis of the S 2*p* spectra, it was found that the broad peak in the 163–164 eV range can be attributed to LiPSs, while the two pairs of peaks around 160 and 160.7 eV correspond to terminal sulfur (S_T_^−1^) and bridging sulfur (S_B_^0^), respectively. Notably, compared with Co-AHF-DPDC, Ni-AHF-DPDC, and AHF-DPDC, the Fe-AHF-DPDC membrane exhibits a significantly weaker LiPSs signal. This indicates that Fe-AHF-DPDC can effectively promote the complete conversion of S_8_ into insoluble Li_2_S_2_ and Li_2_S. Moreover, the stronger signal corresponding to Li_2_S_2_ suggests that the sulfur reduction process is accelerated, highlighting the excellent catalytic performance of Fe-AHF-DPDC in facilitating the redox kinetics of sulfur species.

###  In situ Monitoring of Enhanced Catalytic Reaction Kinetics by Fe-AHF-DPDC in Lithium-Sulfur Batteries

**Fig. 4:**
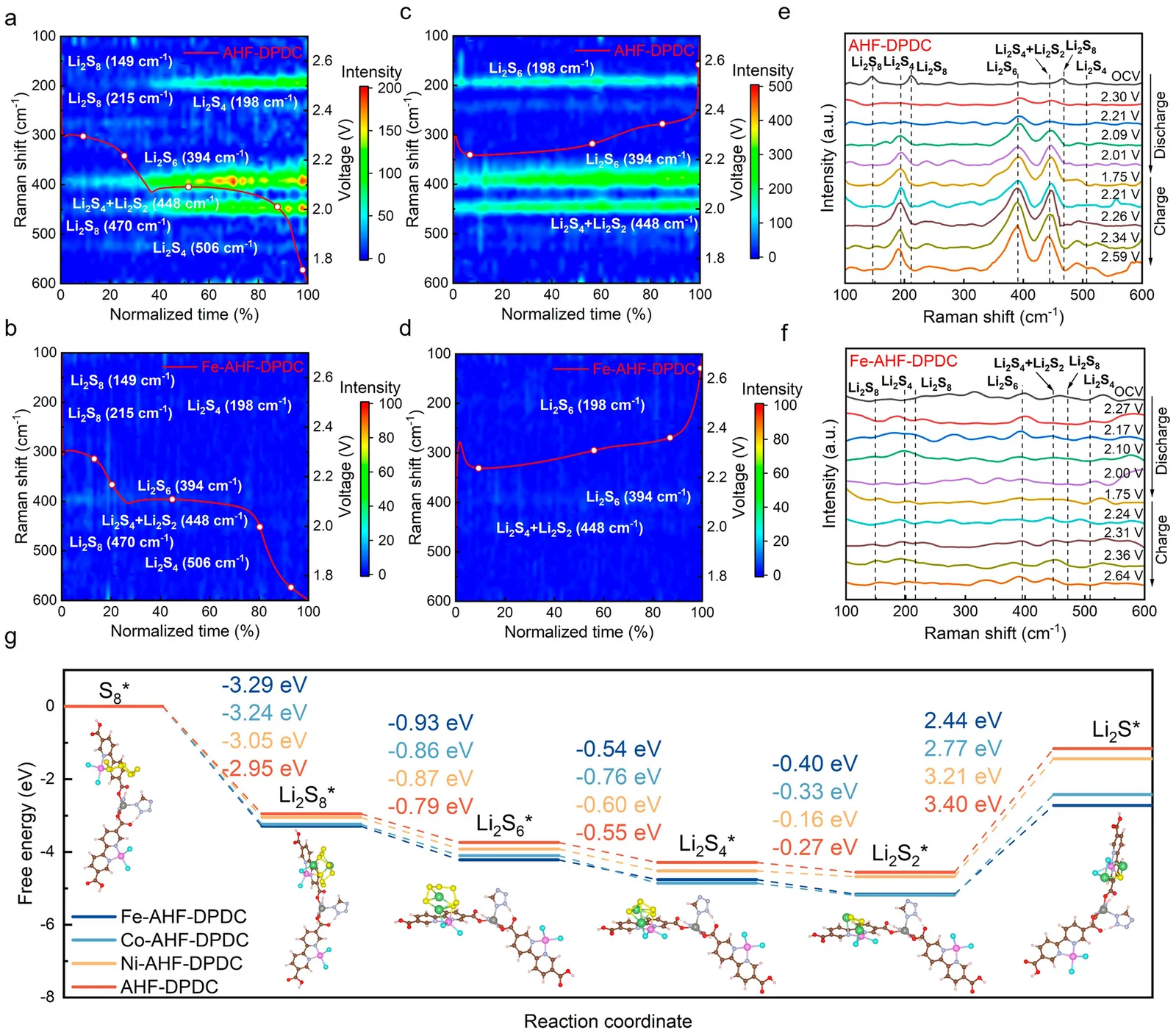
In situ Raman reaction mechanism and theoretical calculations. **a** In situ time-resolved Raman spectra of the AHF-DPDC-modified separator obtained during the discharge process. **b** In situ time-resolved Raman spectra of the Fe-AHF-DPDC-modified separator obtained during the discharge process. **c** In situ time-resolved Raman spectra of the AHF-DPDC-modified separator obtained during the charging process. **d** In situ time-resolved Raman spectra of the Fe-AHF-DPDC-modified separator obtained during the charging process. **e** Raman signals of the Fe-AHF-DPDC-modified separator battery under different voltage states. **f** Raman signals of the AHF-DPDC-modified separator battery under different voltage states. **g** Gibbs free energy curves for LiPSs conversion reactions on AHF-DPDC, Co-AHF-DPDC, Ni-AHF-DPDC, and AHF-DPDC

Systematically investigate the influence of Fe-AHF-DPDC, Co-AHF-DPDC, Ni-AHF-DPDC and AHF-DPDC-modified separators on the transformation behavior of LiPSs and the diffusion mechanisms of sulfur species in LSBs, in situ time-resolved Raman spectroscopy was carried out during the operation of Li–S coin cells at a constant charge/discharge rate of 0.2 C (see Figs. 4a-f, S44, and S45). This technique enables real-time monitoring of chemical changes on the separator side during battery cycling, providing deep insight into how these materials interact with LiPSs in terms of adsorption, catalytic conversion, and suppression of the shuttle effect. As shown in Figs. 4e, 4f and S45e, f, the in situ Raman spectra collected at different discharge/charge stages revealed characteristic peaks corresponding to various sulfur species on the separator side. According to previous literature, these peaks are attributed to LiPSs with different chain lengths: For instance, the peaks at 149, 215, and 470 cm^−1^ correspond to S_8_^2−^ (Li_2_S_8_), the peak at 394 cm^−1^ corresponds to S_6_^2−^ (Li_2_S_6_), and the peaks at 198 and 506 cm^−1^ are assigned to S_4_^2−^ (Li_2_S_4_). The evolution of these signals reflects the generation, transformation, and migration behavior of LiPSs during the charge/discharge process.

Notably, throughout the discharge process, only very weak and slowly fading S_4_^2−^ signals were observed at 198 and 506 cm^−1^ in the Fe-AHF-DPDC-modified separator system. In contrast, the other three separators (Co-AHF-DPDC, Ni-AHF-DPDC, and AHF-DPDC) exhibited significantly enhanced and prolonged LiPSs signals. Characteristic peaks at 198 cm^−1^ (Li_2_S_4_), 394 cm^−1^ (Li_2_S_6_), and 448 cm^−1^ (Li_2_S_2_/Li_2_S_4_) remained detectable throughout the discharge process, indicating that LiPSs in these systems did not fully convert into the final products Li_2_S or Li_2_S_2_. A considerable amount of intermediate species persisted during the reaction, suggesting weaker LiPSs adsorption and catalytic conversion capabilities of these three separators compared to Fe-AHF-DPDC. As a result, LiPSs more easily migrated from the cathode region to the anode side, leading to severe shuttle effects and accelerated capacity decay, which negatively impacted the overall electrochemical performance of the batteries [50].

Taken together, the in situ Raman spectroscopy analysis clearly demonstrates that the Fe-AHF-DPDC-modified separator offers significant advantages in regulating the transformation pathways of LiPSs and suppressing their shuttle behavior. Its superior performance stems not only from the effective physical confinement of LiPSs by the MOF structure’s abundant pore channels and high specific surface area but also from the strong chemical adsorption and catalytic ability provided by the Fe active centers. These combined features enable efficient LiPSs conversion and the formation of a stable interface. The results indicate that Fe-AHF-DPDC possesses both strong physical adsorption and chemical anchoring capabilities toward LiPSs, effectively inhibiting their diffusion into the electrolyte and thereby significantly reducing the likelihood of shuttle effects. Consequently, this leads to enhanced overall electrochemical performance of LSBs [6, 30].

Using density functional theory, the Gibbs free energy curves and stable adsorption configurations of optimized Li_2_S_n_ (1 ≤ n ≤ 8) species during the sulfur reduction reaction (SRR) on Fe-AHF-DPDC, Co-AHF-DPDC, Ni-AHF-DPDC, and AHF-DPDC were calculated, and the corresponding evolution curves were obtained (Fig. 4g). The entire discharge process involves the transformation from S_8_ to Li_2_S. The first step involves the reduction of S_8_ with two Li^+^ atoms to form Li_2_S_8_, which further dissociates into Li_2_S_6_, Li_2_S_4_, and Li_2_S_2_ intermediates, ultimately producing Li_2_S. The breaking of sulfur chains is accompanied by highly exothermic adsorption. During the conversion of Li_2_S_2_ to Li_2_S, the maximum increase in Gibbs free energy is observed, which is considered the rate-determining step of the SRR.

As expected, the Gibbs free energy increase for Li_2_S nucleation at the bimetallic Fe-AHF-DPDC center (2.44 eV) is lower than that at Co-AHF-DPDC (2.77 eV), Ni-AHF-DPDC (3.21 eV), and the monometallic AHF-DPDC (3.40 eV), indicating that the nucleation of Li_2_S on Fe-AHF-DPDC is thermodynamically more favorable. Compared with Co-AHF-DPDC, Ni-AHF-DPDC, and AHF-DPDC, Fe-AHF-DPDC exhibits lower reaction barriers for LiPSs conversion (-3.29, -0.93, -0.54, -0.4, and 2.44 eV), demonstrating that Fe-AHF-DPDC, with its Fe metal centers, possesses superior catalytic capability toward LiPSs, thereby facilitating their rapid transformation.

### Fe-AHF-DPDC Enhances the Performance of Lithium-Sulfur Batteries

This study systematically evaluated the regulatory mechanisms of four functionalized separators on the performance of lithium-sulfur batteries, revealing the advantageous role of Fe-AHF-DPDC materials in multiscale electrochemical processes. As shown in Fig. S46, electrochemical impedance spectroscopy (EIS) analysis indicated that batteries based on Fe-AHF-DPDC exhibited excellent charge transfer characteristics, with a charge transfer resistance (R_ct_ = 16.21 Ω) significantly lower than that of Co-AHF-DPDC (17.22 Ω), Ni-AHF-DPDC (19.41 Ω), and AHF-DPDC (25.32 Ω). The lower interfacial resistance can effectively enhance Li^+^ ion migration kinetics and the rate of redox reactions, thereby promoting the interfacial conversion efficiency of LiPSs [51, 52].

**Fig. 5:**
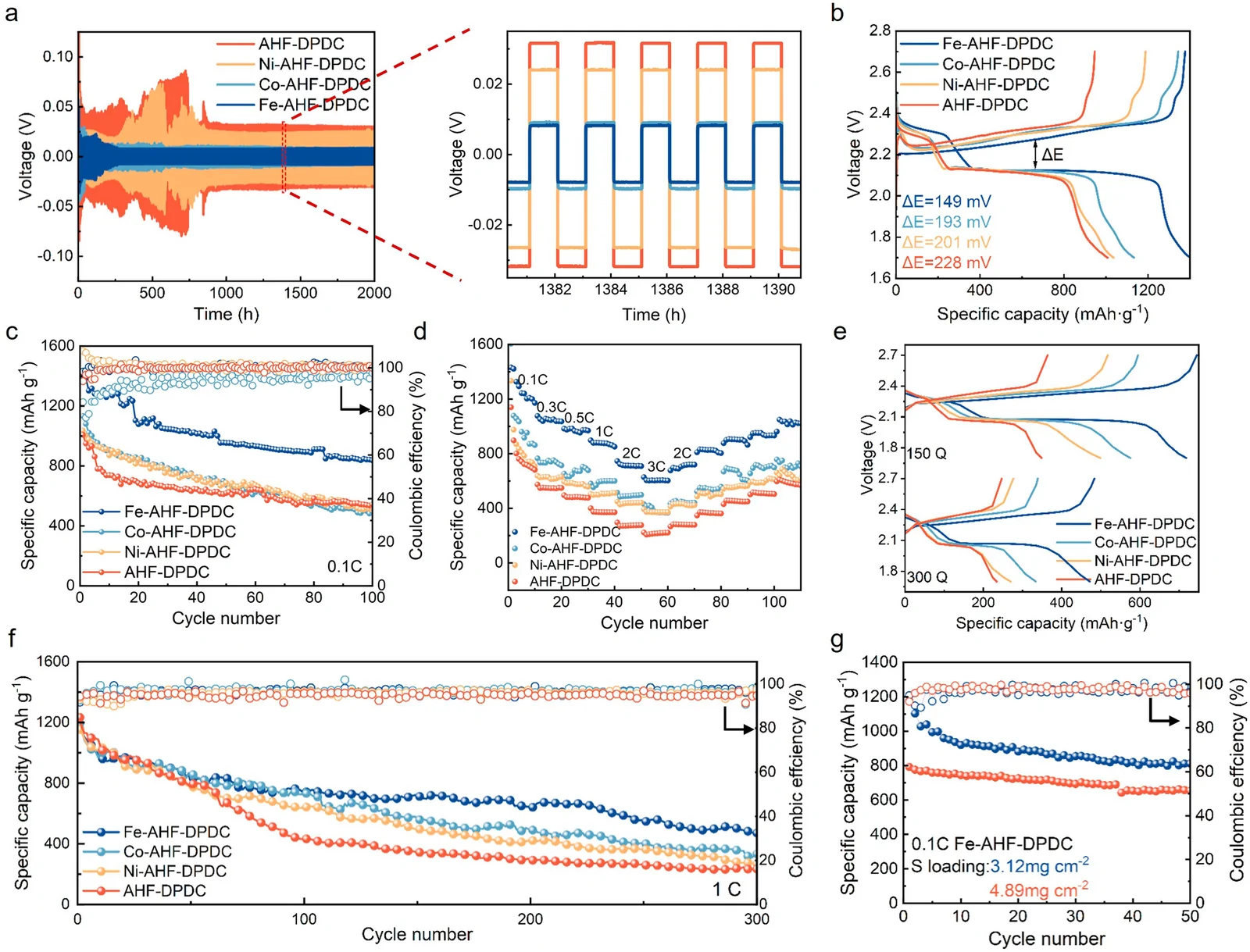
Electrochemical performance of Fe-AHF-DPDC, Co-AHF-DPDC, Ni-AHF-DPDC, and AHF-DPDC Batteries with the 1.0 mg cm^−2^. **a** Cycling stability of Fe-AHF-DPDC, Co-AHF-DPDC, Ni-AHF-DPDC, and AHF-DPDC batteries at a current density of 1 mA cm^−2^ (The inset shows a magnified view of a selected region). **b** Galvanostatic charge–discharge curves of Fe-AHF-DPDC, Co-AHF-DPDC, Ni-AHF-DPDC, and AHF-DPDC batteries at 0.1 C. **c** Cycling performance of Fe-AHF-DPDC, Co-AHF-DPDC, Ni-AHF-DPDC and AHF-DPDC batteries at 0.1 C. **d** Rate capability of Fe-AHF-DPDC, Co-AHF-DPDC, Ni-AHF-DPDC and AHF-DPDC batteries from 0.1 to 3 C. **e** Charge–discharge plateaus of Fe-AHF-DPDC, Co-AHF-DPDC, Ni-AHF-DPDC and AHF-DPDC batteries at 1 C. **f** Long-term cycling performance of Fe-AHF-DPDC, Co-AHF-DPDC, Ni-AHF-DPDC and AHF-DPDC batteries at 1 C. **g** Cycling performance of Fe-AHF-DPDC batteries with cathodes containing 3.12 and 4.89 mg cm^−2^ sulfur loading at 0.1 C

Regarding electrochemical stability, Li//Li symmetric cell tests (Fig. 5a) show that the Fe-AHF-DPDC cell maintains excellent long-term cycling stability for over 2000 h at 1 mA cm^−2^, with the polarization voltage consistently remaining at a low level of 16 mV, far superior to other cells (Co-AHF-DPDC: 18.9 mV; Ni-AHF-DPDC: 26.65 mV; AHF-DPDC: 63.4 mV). These results confirm that the Fe-AHF-DPDC separator can effectively regulate uniform lithium deposition/stripping behavior and significantly suppress the formation of lithium dendrites [29, 53].

Galvanostatic charge–discharge (GCD) curves of Fe-AHF-DPDC, Co-AHF-DPDC, Ni-AHF-DPDC, and AHF-DPDC batteries at 0.1 C (Fig. 5b) showed that during discharge, the higher-voltage plateau corresponds to the reduction of elemental sulfur (S_8_) into soluble polysulfides (e.g., Li_2_S_8_, Li_2_S_6_), which provides a relatively stable specific capacity. The lower voltage plateau corresponds to the further reduction of these soluble polysulfides (e.g., Li_2_S_4_) into insoluble solid sulfides (e.g., Li_2_S_2_ and Li_2_S), which plays a decisive role in the overall specific capacity of the battery. The results show that the Fe-AHF-DPDC separator is able to more effectively promote the sulfur redox reaction (SRR). In addition, the polarization voltage (Fig. S47) of the Fe-AHF-DPDC battery is 149 mV, significantly lower than that of Co-AHF-DPDC (193 mV), Ni-AHF-DPDC (201 mV), and AHF-DPDC (228 mV), suggesting that Fe-AHF-DPDC exhibits less polarization, contributing to improved electrochemical reversibility. In summary, the Fe-AHF-DPDC battery demonstrates higher capacity, indicating its significant advantage in promoting the conversion of soluble polysulfides into insoluble sulfides, thus effectively enhancing the performance of LSBs [54].

The separators of Fe-AHF-DPDC and Co-AHF-DPDC batteries before and after 50 cycles at 0.1 C were characterized by XRD. As shown in Fig. S48, the results indicate that the positions of the diffraction peaks in the XRD patterns remained consistent, and no significant peak shifts or new peaks were observed after cycling. This suggests that the crystal structures of Fe-AHF-DPDC and Co-AHF-DPDC did not undergo noticeable changes or collapse during electrochemical cycling, demonstrating excellent structural stability. Based on this finding, we reasonably speculate that the crystalline materials of Ni-AHF-DPDC and AHF-DPDC would exhibit the same structural stability under similar electrochemical conditions.

The Fe-AHF-DPDC battery exhibits an initial discharge specific capacity of 1400.7 mAh g^−1^ at 0.1 C, significantly higher than Co-AHF-DPDC (1133.4 mAh g^−1^), Ni-AHF-DPDC (1036.1 mAh g^−1^), AHF-DPDC (1008.1 mAh g^−1^), and the unmodified PP separator (820 mAh g^−1^) (Fig. S49). After 100 cycles, the Fe-AHF-DPDC battery still maintains the highest reversible capacity of 847.5 mAh g^−1^ (Figs. 5c and S50). The high discharge specific capacity indicates that Fe-AHF-DPDC can more fully utilize elemental sulfur, contributing to higher energy density [52]. SEM (Figs. S51-S56) analysis of the cycled separators revealed uniform distributions of Fe, Co, and Ni elements in the cross sections of Fe-AHF-DPDC, Co-AHF-DPDC, and Ni-AHF-DPDC separators, respectively, indicating successful chelation of the metal ions into the separators.

As shown in Figs. 5d and S55, the rate performance of Fe-AHF-DPDC, Co-AHF-DPDC, Ni-AHF-DPDC, and AHF-DPDC membranes was tested at different current densities. Fe-AHF-DPDC consistently exhibited higher specific capacity at various charge and discharge rates. At current densities of 0.1, 0.3, 0.5, 1, 2, and 3 C, the initial discharge specific capacities of the Fe-AHF-DPDC battery were 1431.9, 1076.8, 983.9, 898.1, 745.7, and 628.9 mAh g^−1^, respectively. When the current density returned to 0.1 C, the discharge specific capacity recovered to 977.9 mAh g^−1^, outperforming Co-AHF-DPDC (1604.8, 766.3, 654.3, 581.6, 514.5, and 431 mAh g^−1^), Ni-AHF-DPDC (1334.8, 638.8, 595.1, 504.8, 449.8, and 393.9 mAh g^−1^), and AHF-DPDC (1140.3, 574.1, 496.6, 400.9, 296.5, and 223.6 mAh g^−1^). This indicates that the Fe-AHF-DPDC-modified separator can not only effectively promote the conversion reactions of LiPSs during multiple cycles and high-rate charging and discharging but also significantly reduce energy losses in the redox process, thereby improving the battery’s efficiency and overall performance. The charge–discharge voltage curves at different current densities (Fig. S56) show that, compared with Co-AHF-DPDC, Ni-AHF-DPDC, and AHF-DPDC separators, the Fe-AHF-DPDC separator can maintain lower voltage polarization even at high current densities. This phenomenon confirms its significant enhancement of the LiPSs redox reaction kinetics, as well as its improved electronic conductivity and ionic diffusion capability [55].

The long-term stability of Fe-AHF-DPDC batteries was evaluated at 1 C. As shown in Figs. 5f and S56, the initial capacities of Fe-AHF-DPDC, Co-AHF-DPDC, Ni-AHF-DPDC, and AHF-DPDC were 1157.3, 1195.3, 1153.8, and 1234.2 mAh g^−1^, respectively. After 300 cycles, the capacities decreased to 471.9, 333.8, 269.8, and 234 mAh g^−1^, while the coulombic efficiency remained close to 100%. Comparing the charge–discharge curves of the four LSBs at the 150th and 300th cycles (Fig. 5e), the discharge platform indicates that Fe-AHF-DPDC exhibits better cycling reversibility than Co-AHF-DPDC, Ni-AHF-DPDC, and AHF-DPDC. This superior performance is mainly attributed to the unique physicochemical properties of the Fe-AHF-DPDC material. On one hand, the MOF structure possesses abundant pore channels and a high specific surface area, which can effectively adsorb LiPSs and suppress the shuttle effect. On the other hand, the presence of Fe active sites enhances catalytic capability toward LiPSs, accelerating their conversion kinetics during charge–discharge processes, allowing sulfur to be more efficiently converted into final products Li_2_S/Li_2_S_2_ during discharge [56].

Under conditions where the sulfur loading on the cathode was 3.12 (E/S = 7.8 μL cm^−1^) and 4.89 mg cm^−2^ (E/S = 8 μL cm^−1^) (Figs. 5g and S57), Fe-AHF-DPDC batteries delivered initial specific capacities of 1174.6 and 791.9 mAh g^−1^ at 0.1 C, respectively. After 50 cycles, the capacities remained at 811.6 and 654.1 mAh g^−1^, with retention rates of 69.1% and 82.6%, respectively. Furthermore, compared with existing related studies, this study demonstrates significant performance advantages (Fig. S58). To visually demonstrate the safety of Fe-AHF-DPDC pouch cells (Fig. S59), Fe-AHF-DPDC separators were assembled into pouch cells and subjected to bending (> 90°), cutting, and puncture tests. The small light bulb remained lit continuously, and the pouch cells showed no significant swelling. These results indicate that integrating the Fe-AHF-DPDC cathode material into LiS pouch cells holds great potential for wearable device applications.

## Conclusions

In summary, we successfully Li^+^ designed and synthesized a novel series of one-dimensional macroporous M-AHF-DPDC (M = Fe, Co, Ni) crystals featuring a dual-transport mechanism that simultaneously enables selective sieving and catalyzes the conversion of dissolved polysulfides in LSBs. Incorporation of Fe centers into the MOF framework significantly enhances catalytic activity toward polysulfide redox reactions compared to pristine AHF-DPDC, leading to superior electrochemical performance, including a high initial capacity of 1157.35 mAh g^−1^ at 1 C, 1400.7 mAh g^−1^ at 0.1 C, and stable cycling over 700 cycles. The Fe-AHF-DPDC-modified separator also maintains excellent performance under challenging conditions of high sulfur loading (4.89 mg cm^−2^) and lean electrolyte (E/S = 8 μL cm^−1^). Furthermore, Li||Li symmetric cells employing the Fe-AHF-DPDC separator demonstrate uniform Li^+^ deposition and outstanding long-term cycling at 1 mA cm^−2^, collectively validating the unique molecular sieving and catalytic functionality of this design. These results reveal a synergistic mechanism combining polysulfide blocking and catalytic conversion, effectively suppressing the shuttle effect while ensuring homogeneous Li^+^ transport, offering a feasible strategy for high-performance, high-energy–density rechargeable lithium–sulfur batteries.

## Supplementary Information

Below is the link to the electronic supplementary material.

Supplementary file1 (DOCX 15205 KB)
