# Supplementary material for: Single-Metal-Anchored 1D Mesoporous Channels to Enable Accelerated Redox Kinetics for Lithium-Sulfur Batteries
Source: Nanomicro Lett. 2026 Apr 21;18:340. doi: 10.1007/s40820-026-02177-w (PMC13100228; doi:10.1007/s40820-026-02177-w)
Supplement: Supplementary file 1 — Supplementary file1 (DOCX 15205 KB) [file 40820_2026_2177_MOESM1_ESM.docx]

Supplementary Information for

**Single-Metal-Anchored 1D** **Mesoporous Channels to Enable Accelerated Redox Kinetics for Lithium-Sulfur Batteries**

Dequn Zhao^1, 2#^, Shun Wang^1, 2#^, Yanan Zhang^1, 2*^, Xingxing Zhang^2, 3^, Xuehan Hou^1, 2^, Hong Wang^4^, Xiangyu Liu*^5^*^*^*,* Feiyang Yin^6^, Wei Zhou^6*^, Wenhuan Huang^1, 2, 4*^

^1^ Key Laboratory of Chemical Additives for China National Light Industry, College of Chemistry and Chemical Engineering, Shaanxi University of Science and Technology, Xi’an 710021, P. R. China

^2^ Flexible Energy storage and Interfacial Chemistry Key Laboratory of Shaanxi University, College of Chemistry and Chemical Engineering, Shaanxi University of Science and Technology, Xi’an 710021, P. R. China

^3^ Xi’an Key Laboratory of Advanced photo-electronics Materials and Energy Conversion Device, Technological Institute of Materials & Energy Science (TIMES), Xijing University, Xi’an 710123, P. R. China

^4^ Shanghai Lingfang Energy Co. Ltd., Shanghai 201100, P. R. China

^5^ State Key Laboratory of High-Efficiency Utilization of Coal and Green Chemical Engineering, College of Chemistry and Chemical Engineering, Ningxia University, Yinchuan 750021, P. R. China

^6^ School of Chemistry and Chemical Engineering, Hainan University, 58 Renmin Avenue, Haikou, 570228, P. R. China

^#^ Dequn Zhao and Shun Wang contributed equally to this work.

*Corresponding author. E-mail: [zhangyanan@sust.edu.cn](mailto:zhangyanan@sust.edu.cn) (Yanan Zhang); [xiangyuliu432@126.com](mailto:xiangyuliu432@126.com) (Xiangyu Liu); [weichou2023@hainanu.edu.cn](mailto:weichou2023@hainanu.edu.cn) (Wei Zhou); [huangwenhuan@sust.edu.cn](mailto:huangwenhuan@sust.edu.cn) (Wenhuan Huang)

**S1 Experimental Procedures**

**S1.1 Materials**

All chemical reagents are analytical grade and do not require purification prior to use. FeCl_2_·4H_2_O, CoCl_2_·6H_2_O, NiCl_2_·6H_2_O, Tetrazole, 2,2'-Bipyridine-5,5'-dicarboxylic acid (DPDC), 4, 4′-biphenyldicarboxylic acid (BPDC), N-methyl-2-pyrrolidone (NMP, AR, ≥99.0%), N,N-dimethylacetamide (DMA), Tetrapropylammonium hydroxide solution (TBAOH), polypropylene (PP) separator (*Celgard*^TM^ 2500) were obtained from Shanghai Aladdin Biochemical Technology Co., Ltd. Carbon nanotubes (CNT), 1,2-dimethoxyethane (DME) and 1,3-dioxolane (DOL), Polyvinylidene fluoride (PVDF, average Mn ~1000000), Conductive carbon black (SuperP Li), aluminum (Al) foil, lithium (Li) foil and bis(trifluoromethanesulfony)imide (LiTFSI) were purchased from Shanghai Qing song New Energy Ltd. lithium sulfides (Li_2_S) and sulfur (S) were obtained Sinopharm Chemical Reagent Co. Ltd.

**S1.2 Preparation of Fe-DPDC, Co-DPDC and Ni-DPDC**

1 mmol of 2,2'-bipyridine-5,5'-dicarboxylic acid and 1 mmol of FeCl_2_·4H_2_O powder were weighed and added to a reactor containing 50 mL of deionized water, respectively. After stirring at 600 rpm for 3 days at room temperature, the mixture was filtered, washed three times with 20 mL of deionized water each time, and dried to obtain a light-yellow powder designated as Fe-DPDC. For the preparation of pink Co-DPDC powder and green Ni-DPDC powder, all steps remained the same except for replacing FeCl_2_·4H_2_O with CoCl_2_·6H_2_O and NiCl_2_·6H_2_O powders.

**S1.3 Preparation of AHF-DPDC, Fe-AHF-DPDC, Co-AHF-DPDC and Ni-AHF-DPDC**

Briefly, 1 mmol of 2,2'-bipyridine-5,5'-dicarboxylic acid and 1 mmol of 1H-tetrazole powder were weighed and added to a reactor containing 14 mL of DMA. The solution was stirred at room temperature until homogeneous. Then, 1 mmol of zinc nitrate hexahydrate was added and stirred until completely dissolved. Subsequently, an appropriate amount of 4-methylammonium hydroxide solution was added to adjust the pH to a range of 6.7 ± 0.2. The reaction mixture was then heated to 120 °C for 2 days. After cooling to room temperature, the mixture was filtered and washed three times with 10 mL of DMAc each time. The AHF-DPDC crystals were obtained after drying. To prepare Fe-AHF-DPDC, Co-AHF-DPDC, or Ni-AHF-DPDC, all steps remained the same except for replacing DPDC with Fe-DPDC, Co-DPDC, or Ni-DPDC powder.

**S1.4 Synthesis of Fe-AHF-DPDC, Co-AHF-DPDC, Ni-AHF-DPDC and AHF-DPDC Modified Separators**

The Fe-AHF-DPDC, conductive carbon black (SuperP Li) and polyvinylidene fluoride (PVDF) were mixed in a mass ratio of 7:2:1. The mixture was ground in a mano mortar for 30 minutes. After thorough grinding, a suitable separator of N-methyl-pyrrolidone solution was added to the mortar to form a uniform thick black slurry. The commercial separator was cleaned with anhydrous ethanol and the resulting paste was evenly coated on its surface with a thickness of 100 nm using the scraping coating method. It was then transferred to a vacuum drying oven and dried at 60 ℃ for 12 h. After removal from the oven, it was cut into a round sheet with a diameter of 19 mm using a cutting machine to obtain the modified separator. For the preparation of Co-AHF-DPDC, Ni-AHF-DPDC, or AHF-DPDC septums, all steps remain the same except for the addition of Co-AHF-DPDC, Ni-AHF-DPDC, or AHF-DPDC powder.

**S1.5 Synthesis of Sulfur Cathodes**

The carbon nanotube/sulfur (CNT/S) composites were prepared by a melt diffusion method. First, carbon nanotubes were mixed with sulfur powder in a ratio of 3:1 by weight, homogeneously ground and then heated at 155 °C for 12 h. A sulfur cathode slurry was prepared by mixing the resulting CNT/S composite, SuperP Li and binder PVDF in a ratio of 7:2:1. The slurry was applied to aluminum foil and vacuum dried at 60°C to obtain a sulfur cathode.

**S1.6 Electrochemical Characterization**

The sulfur cathode was prepared by mixing the active materials, SuperP Li, and PVDF (5 wt% PVDF in NMP) in a weight ratio of 7:2:1. Homogeneous slurry was cast onto an aluminum foil, and dried at 60 °C for more than 12 h. Finally, the sulfur cathode was punched into disks with 14 mm diameter. CR2032 typed Li-S coin cells were assembled in an Ar-filled glovebox with the contents of water and oxygen both lower than 0.1 ppm. The Li foil with a thickness of 450 μm was selected as the anode and 50 μL electrolyte with 1 mol L^-1^ LiTFSI in 1, 2-dimethoxyethane (DME) and 1,3-dioxolane (DOL) (1:1, by volume) in the coin cell.

**S1.7 Characterization**

X-ray diffraction (XRD) patterns (Bruker D2 Phaser X-ray Diffractometer with Ni filtered Cu Kα radiation). were used to confirm the crystal structure. The morphology of these samples was observed using a scanning electron microscope (SEM FEI Verios 460) equiped with energy-dispersive X-ray spectroscopy (EDS). The X-ray photoelectron spectroscopy (XPS) analysis was carried out by ESCALAB 250 Xi spectrometer (VG Scientific Co., UK), and all the binding energies obtained in XPS spectra were calibrated using the C 1*s* peak at 284.6 eV. FTIR spectra were collected by 3 a Bruker Alpha P spectrometer with reflection ATR module in emission from 4000 to 400 cm^−1^. In Situ Raman Spectroscopy Measurements Li–S coin cells with a quartz window on the positive shell were used for in situ Raman spectroscopy. A 532 nm Raman laser (LabRam HR Evolution, Horiba, Japan) was used to excite the cathode materials on the Al grid. The batteries were discharged at 0.2 C, and the Raman signals were acquired every 5 min.

**S1.8 Electrochemical Test**

The galvanostatic charge-discharge tests were performed on the LAND CT3002A battery test system, the voltage range is 1.7-2.7 V (vs. Li^+^/Li). The tests of cyclic voltammetry (CV) and electrochemical impedance spectroscopy (EIS) are completed on the electrochemical workstation, and the frequency range of the test is 100 kHz~0.01 Hz. All electrochemical tests are done at a constant temperature of 25 °C. The galvanostatic charge discharge and GITT tests were measured on the LAND CT3002A test system with a voltage range of 1.7-2.7 V.

**S1.9 Ionic Conductivity**
To measure the lithium-ion conductivity, each separator wetting the electrolyte is sandwiched between two stainless steel electrodes and assembled into a coin cell. The ionic conductivity of the MOF modified separators was calculated through an EIS method. The ionic conductivity was calculated according to the following equation (S1):

$\sigma=\frac{d}{SR_{b}}$ (S1)

where *d* (cm) was the thickness of the solid electrolyte, *R_b_* (Ω) and *S* (cm^2^) represented the bulk resistance and the effective area of the solid electrolyte, respectively, and *σ* is the ionic conductivity (S cm^-1^).

**S1.10 Lithium-Ion Transference Number**

The lithium transference number (*t_Li_^+^*) was obtained by using AC impedance and DC potentiostatic polarization measurements with Li//Li symmetric cell. A DC potential (*ΔV*=10 mV) was applied for 2000 s to gain the initial and steady currents. Meanwhile, the AC impedance spectra of the same cell were measured before and after polarization. The value of *t*_Li_^+^ was been calculated by equation (S2):

$t_{{Li}^{+}}=\frac{I_{S}\left( \Delta V-I_{0}R_{0} \right)}{I_{0}\left( \Delta V-I_{S}R_{S} \right)}$ (S2)

Where *R_0_* and *R_s_* are the AC impedances before and after polarization, respectively. *I_0_* and *I_s_* are the initial and steady currents respectively.

**S1.11 Lithium-Ion Diffusion Coefficient**

To measure the lithium-ion conductivity, each separator wetting the electrolyte is sandwiched between two stainless steel electrodes and assembled into a coin cell. The ionic conductivity of the MOF modified separators was calculated through an EIS method. ionic conductivity was obtained according to the following equation (S3):

$I_{P}=(2.69\times{10}^{5})n^{0.5}AD_{{Li}^{+}}^{0.5}C_{{Li}^{+}}v^{0.5}$ (S3)

where *I_p_* (A) peak current value, n represents the reaction of the number of electrons (Li-S battery, *n*=2), *A* (cm^2^) represents the electrode area (cm^2^), *C_Li_^+^* (mol mL^-1^) means the concentration of lithium ions in the electrolyte, and v represents the scan rate (mV s^-1^).

**S1.12 Nucleation Test of Li_2_S**

The Li_2_S_8_ stock solution was prepared by combining sulfur and Li_2_S powder at a molar ratio of 7:1 in tetraglyme under vigorous stirring for 24 h, which was used as the electrolyte. The electrode material (Fe-AHF-DPDC, Co-AHF-DPDC, Ni-AHF-DPDC and AHF-DPDC), SuperP Li and PVDF binder with a mass ratio of 7:2:1 was dispersed in NMP. The resultant slurry was coated on aluminum foil using a blade. The electrode disks with a diameter of 14.0 mm were punched after thorough drying. These disks were used as theidentical working and lithium metal as the counter electrodes. The mass loading of active material was ~1.0 mg cm^2^. 20 μL of Li_2_S_8_ catholyte was dropped on the side of Fe-AHF-DPDC, Co-AHF-DPDC, Ni-AHF-DPDC or AHF-DPDC working electrode, while 20 μL electrolyte without Li_2_S_8_ was added to the side of counter electrode. The assembled cells were discharged galvanostatically to 2.06 V at 112 µA and then discharged potentiostatically to 2.05 V for Li_2_S nucleation/growth.

**S1.13 Shuttle Currents of the Li||S cells with Fe-AHF-DPDC, Co-AHF-DPDC, Ni-AHF-DPDC and AHF-DPDC.**

Shuttle currents of the Li||S cells with Fe-AHF-DPDC, Co-AHF-DPDC, Ni-AHF-DPDC or AHF-DPDC. were measured. Before the shuttle current test, the cell was galvanostatically charged and discharged at 0.1 C for 3 cycles, and then the cells were galvanostatically charged to the 100 % state at 2.7 V, followed by discharging to different voltage stages at 2.70, 2.60, 2.50, 2.45, 2.40, 2.35, 2.30, 2.25 and 2.20 V, and then switched to potentiation mode. The shuttle current was a potentiostatic steadystate current at different voltage levels.

**S1.14 CV Tests of Symmetric Cells**

Electrodes composed of 70 wt% Fe-AHF-DPDC, Co-AHF-DPDC, Ni-AHF-DPDC or AHF-DPDC, 20 wt% SuperP Li, and 10 wt% PVDF for symmetric cells tests were prepared in the same way as electrodes for LSBs. In detail, two pieces of the above electrode were used as the working and counter electrode, and 40 μL of electrolyte containing 0.25 M Li_2_S_6_ solution was added to each cell. Thereafter, the CV profiles of symmetric cells were recorded at a scan rate of 10 mV s^-1^ in the range of -1.0 and 1.0 V.

**S1.15 DFT calculations**

Density functional theory (DFT) calculations were carried out using the Vienna *Ab initio* Simulation Package (VASP) [S1, S2]. The generalized gradient approximation (GGA) proposed by Perdew, Burke, and Ernzerhof (PBE) was adopted to treat the exchange–correlation effects [S3], while the projector augmented wave (PAW) method was employed to represent the electron–ion interactions [S4]. A kinetic energy cutoff of 400 eV was applied throughout the calculations. For Brillouin zone sampling, a Γ-centered Monkhorst–Pack k-point mesh of 1×1×1 was used. The self-consistent field (SCF) procedure was considered converged when the total energy difference between iterations was below 1×10^-4^ eV and the residual force on each atom was less than 0.03 eV Å^-1^. In addition, long-range dispersion forces were taken into account by the DFT-D3 correction scheme [S5].

**Supplementary Figures and Tables**


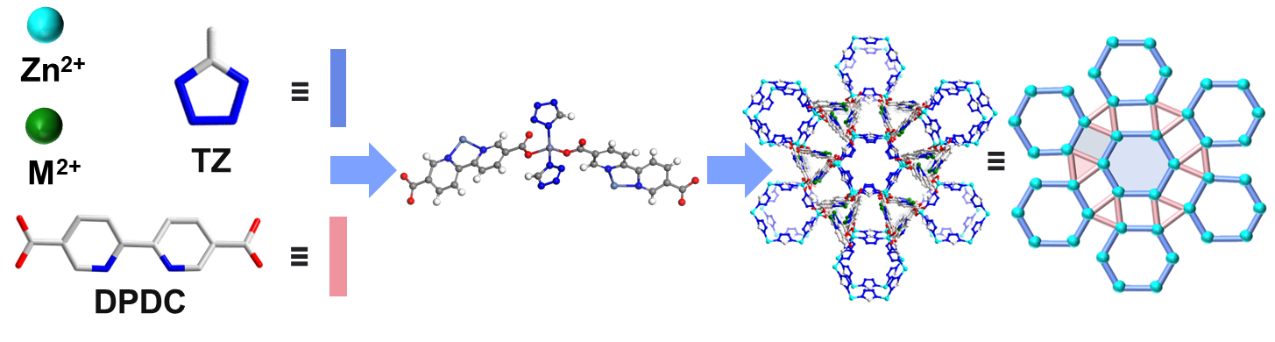


**Fig. S1** Structural units and pore structure of M-AHF-DPDC (M=Fe, Co, Ni) framework


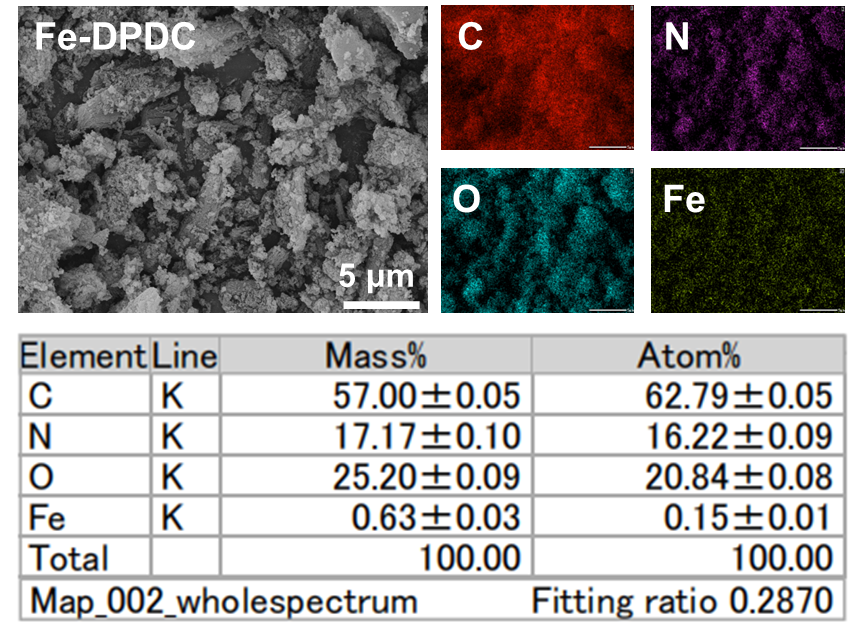


**Fig. S2** SEM images of Fe-DPDC with corresponding C, N, O, and Fe element mappings


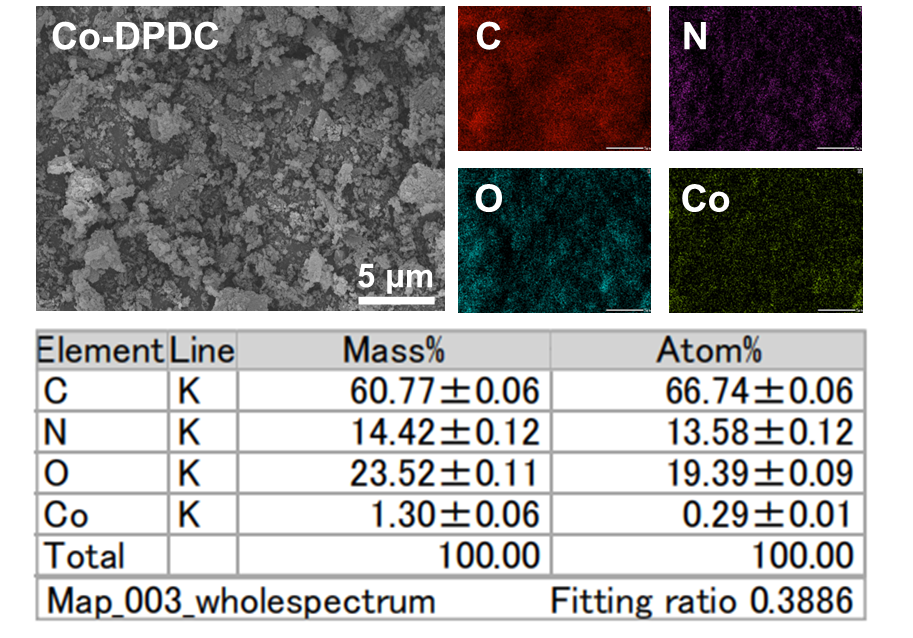


**Fig. S3** SEM images of Co-DPDC with corresponding C, N, O and Co element mappings


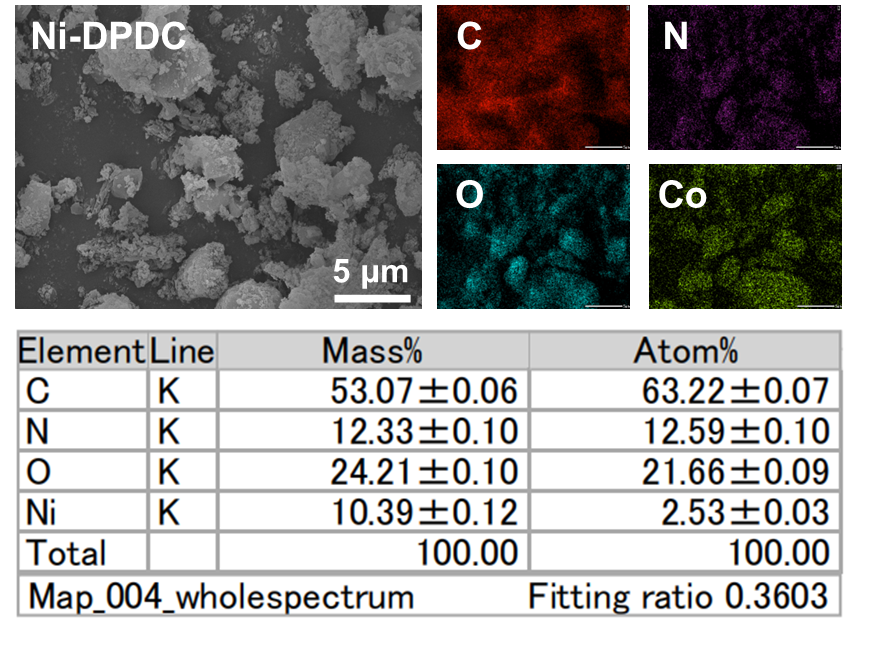


**Fig. S4** SEM images of Ni-DPDC with corresponding C, N, O and Ni element mappings


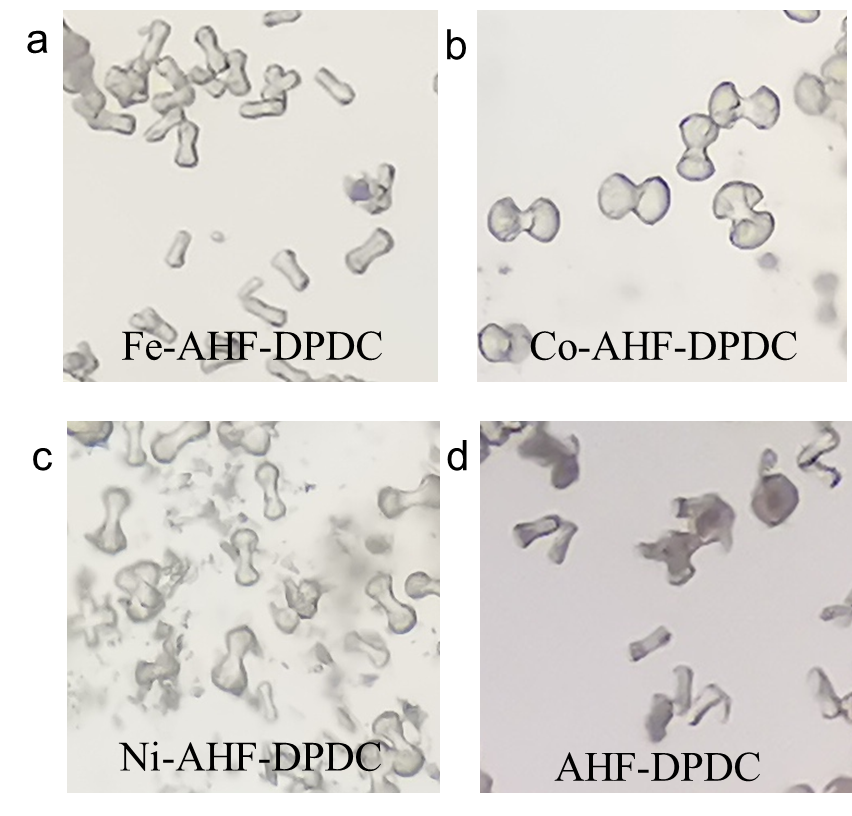


**Fig. S5** Light microscope Optical photos of the synthesized Fe-AHF-DPDC (**a)**, Co-AHF-DPDC (**b)**, Ni-AHF-DPDC (**c)**, and AHF-DPDC (**d)**


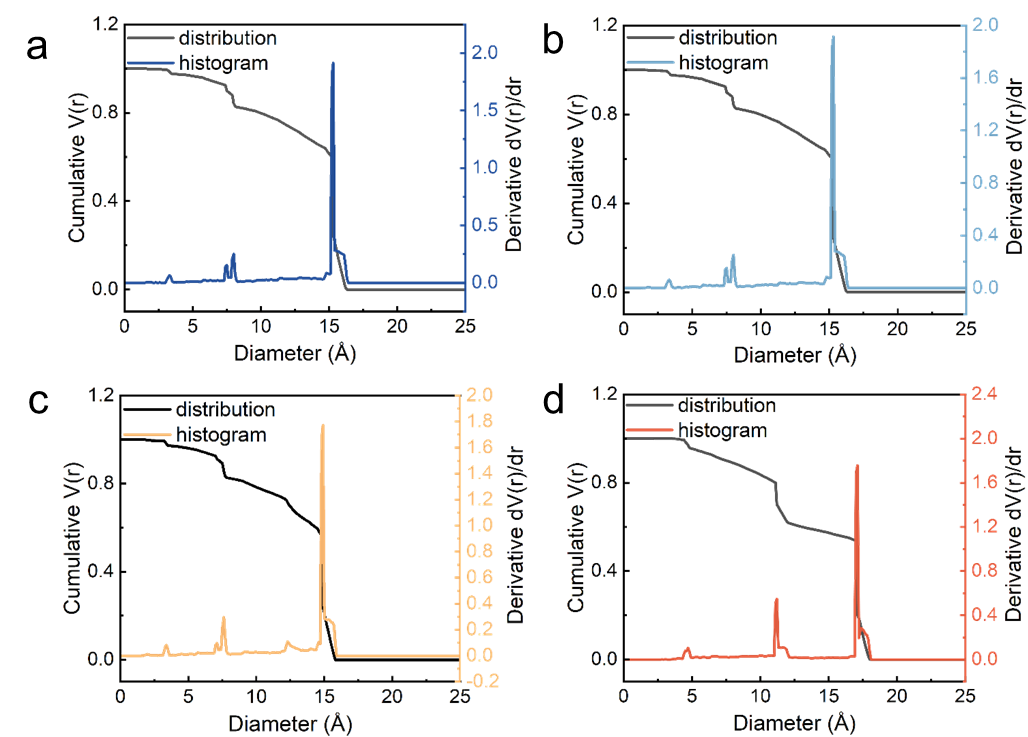


**Fig. S6** Apertures of Fe-AHF-DPDC (**a**), Co-AHF-DPDC (**b**), Ni-AHF-DPDC (**c**) and AHF-DPDC (**d**) based on Zeo++ software


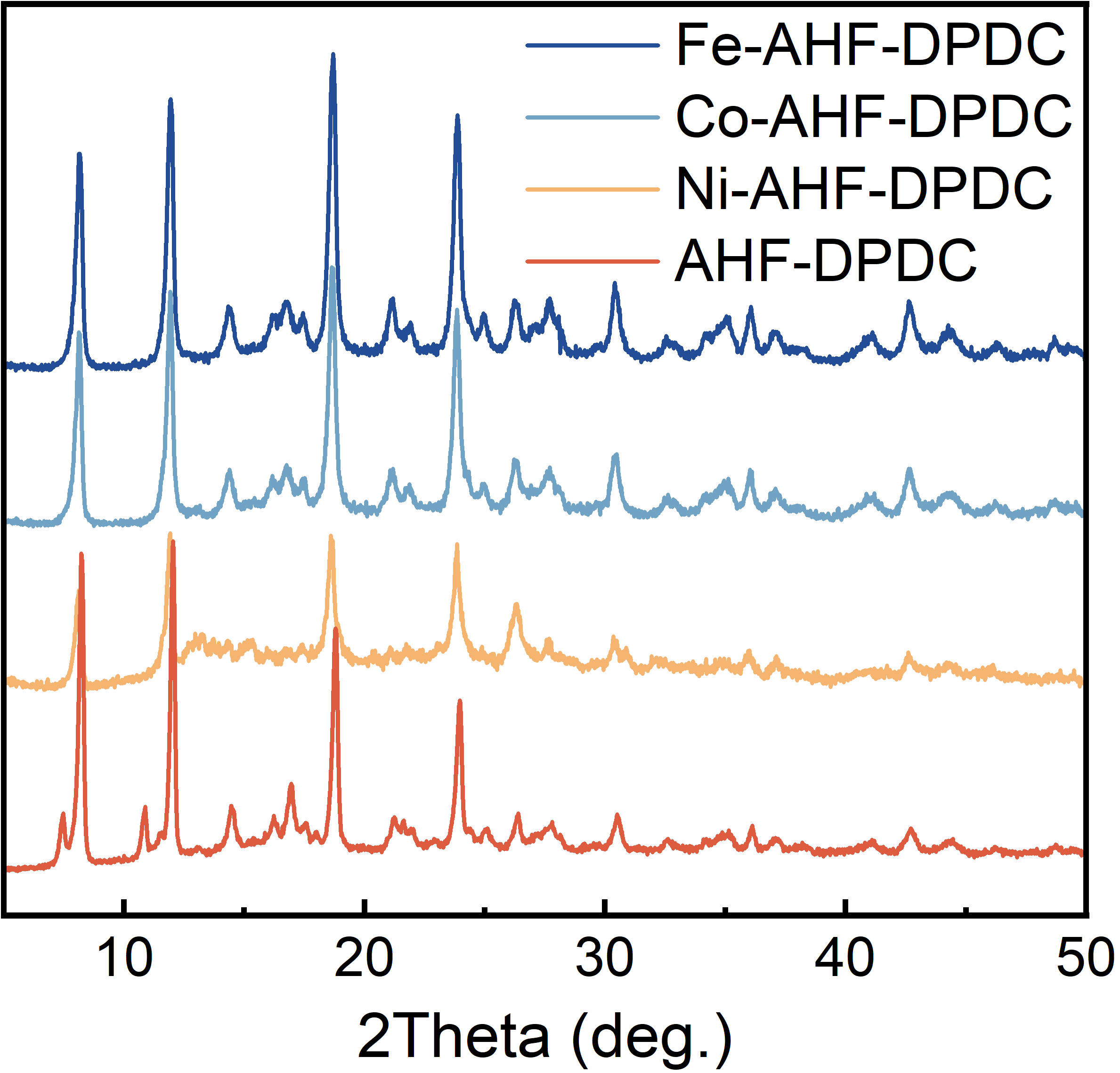


**Fig. S7** XRD patterns of MOF particles


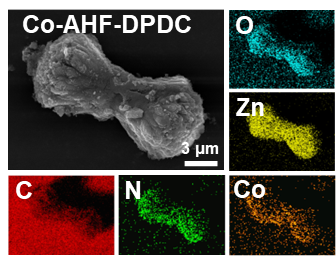


**Fig. S8** SEM and Mapping images of Co-AHF-DPDC


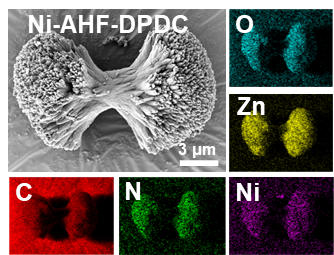


**Fig. S9** SEM and Mapping images of Ni-AHF-DPDC


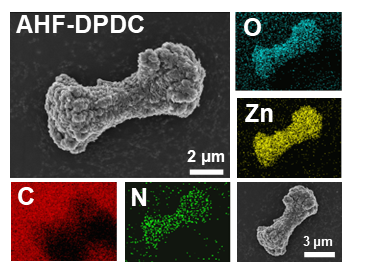


**Fig. S10** SEM and Mapping images of AHF-DPDC


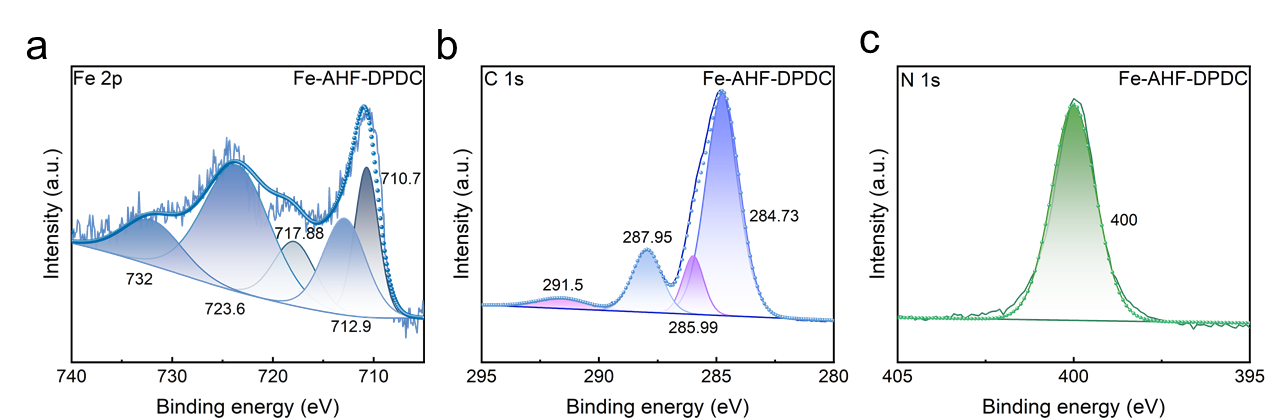


**Fig. S11** XPS spectra of Fe-AHF-DPDC, high-resolution spectra of Fe 2*p* (**a**), C 1*s* (**b**) and N 1*s* (**c**) respectively. (Fe²⁺ 2*p₃/₂*) and 723.6 eV (Fe²⁺ 2*p₁/₂*), corresponding to Fe²⁺ species, while the characteristic peak for Fe³⁺ species appears at 712.9 eV (Fe³⁺ 2*p₃/₂*). Additionally, the peaks observed at 717.88 and 732 eV can be attributed to satellite peaks of Fe 2*p*, indicating that partial oxidation of Fe²⁺ to Fe³⁺ occurs during the coordination process. Furthermore, the C 1*s* spectrum exhibits distinct characteristic peaks at 284.73, 285.99, and 287.95 eV, which are assigned to C–N, C–O, and C=O bonds, respectively, further confirming the successful incorporation of organic ligands into the material


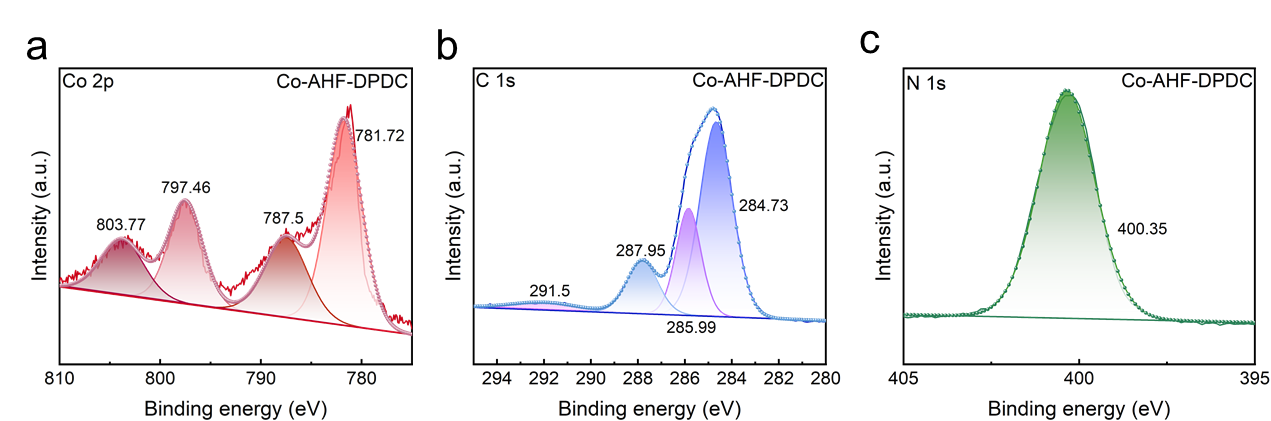


**Fig. S12** XPS spectra of Co-AHF-DPDC, high-resolution spectra of Co 2*p* (a), C 1*s* (**b**) and N 1*s* (**c**) respectively. XPS analysis was performed on Co-AHF-DPDC. The Co 2*p* spectrum can be deconvoluted into four characteristic peaks: the peaks at 781.72 and 797.46 eV are assigned to Co 2*p₃/₂* and Co 2*p₁/₂*, respectively, while the peaks at 787.5 and 803.77 eV correspond to their satellite peaks. These binding energy values indicate that cobalt in Co-AHF-DPDC primarily exists in the Co³⁺ oxidation state


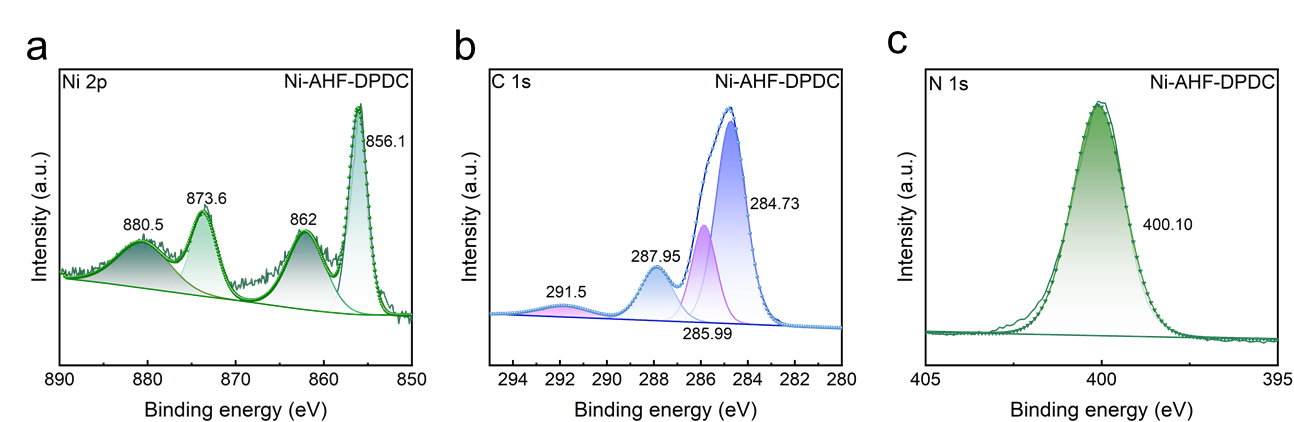


**Fig. S13** XPS spectra of Co-AHF-DPDC, high-resolution spectra of Co 2*p* (**a**), C 1*s* (**b**) and N 1*s* (**c**) respectively. The high-resolution XPS spectrum of Ni shows two main peaks at 856.1 and 873.6 eV, corresponding to the Ni 2*p₃/₂* and Ni 2*p₁/₂* levels, respectively, while the peaks at 862 and 880.5 eV are attributed to satellite peaks. These binding energy values indicate that nickel in Ni-AHF-DPDC primarily exists in the Ni²⁺ oxidation state


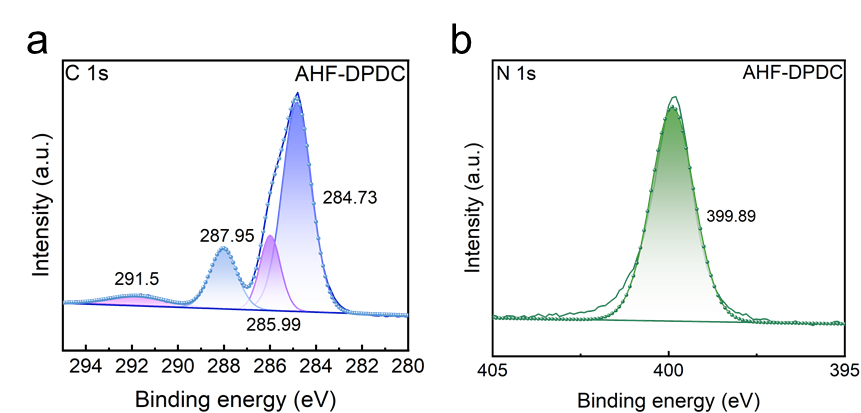


**Fig. S14** XPS spectra of Ni-AHF-DPDC, high-resolution spectra of C 1*s* (**a**) and N 1*s* (**b**) respectively


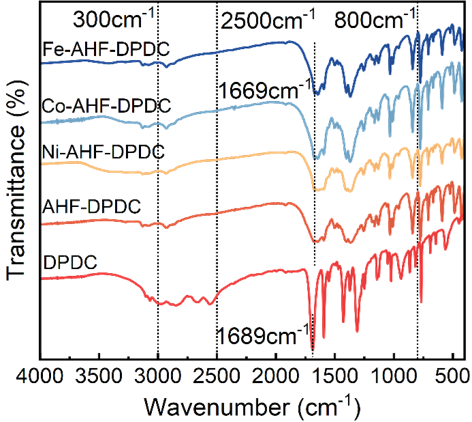


**Fig. S15** Corresponding FI-IR patterns for Fe-AHF-DPDC, Co-AHF-DPDC, Ni-AHF-DPDC, AHF-DPDC and DPDC


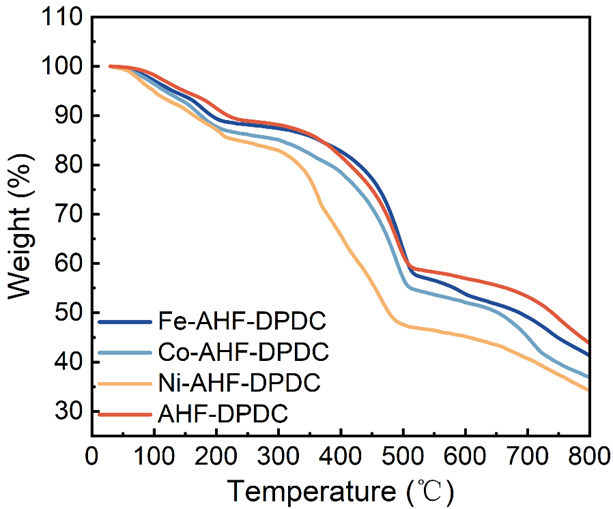


**Fig. S16** Thermogravimetric analysis (TGA) curves of Fe-AHF-DPDC, Co-AHF-DPDC, Ni-AHF-DPDC, and AHF-DPDC. The figure presents the thermogravimetric analysis (TGA) curves of four materials: Fe-AHF-DPDC, Co-AHF-DPDC, Ni-AHF-DPDC, and AHF-DPDC, revealing their mass change trends at different temperatures. It can be observed from the figure that these four materials exhibit similar weight-loss behaviors during the heating process, although there are slight differences at specific temperature points. The weight loss below 150 °C is attributed to the evaporation of moisture. All four MOFs begin to undergo thermal decomposition around 350 °C, where the weight loss rate significantly increases, indicating the breakdown of organic ligands. When the temperature reaches 520 °C, the weight loss process stabilizes, suggesting that the main framework structure may have transformed into inorganic residues [S6]. Overall, Fe-AHF-DPDC, Co-AHF-DPDC, and Ni-AHF-DPDC exhibit slightly higher thermal stability than AHF-DPDC, which may be attributed to the enhanced structural stability provided by the metal ions


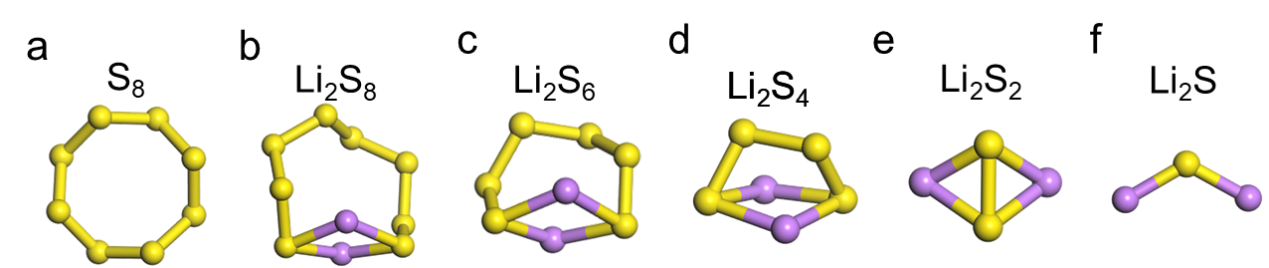


**Fig. S17** The structures of (**a**) S_8_, (**b**) Li_2_S_8_, (**c**) Li_2_S_6_, (**d**) Li_2_S_4_, (**e**) Li_2_S_2_ and (**f**) Li_2_S. Purple balls represent Li atoms. Yellow balls represent S atoms


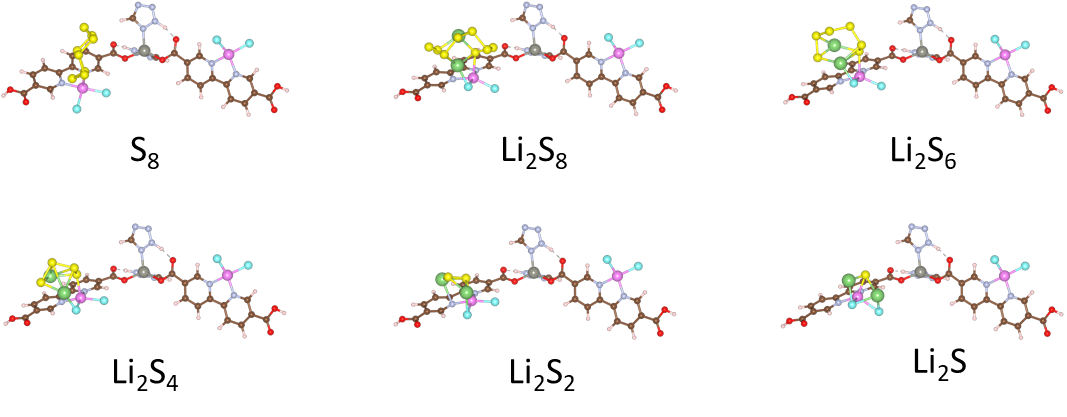


**Fig. S18** Optimal structural model for adsorption of polysulfides by Fe-AHF-DPDC


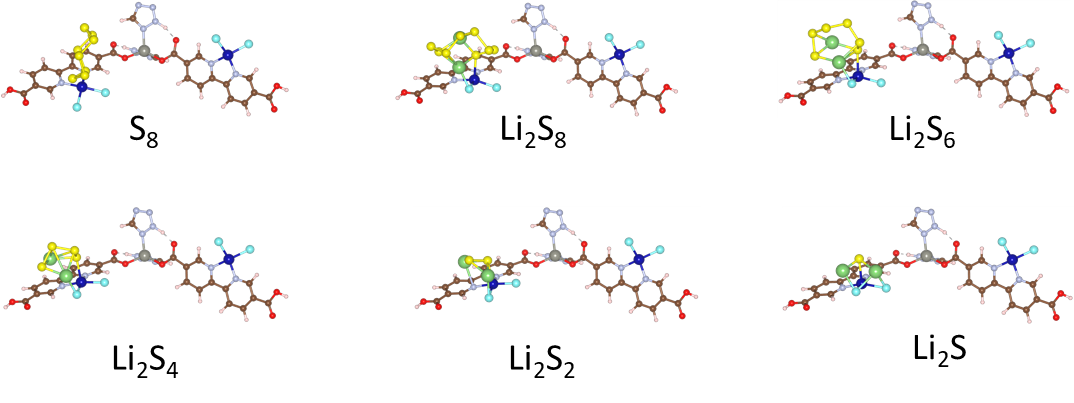


**Fig. S19** Optimal structural model for adsorption of polysulfides by Co-AHF-DPDC


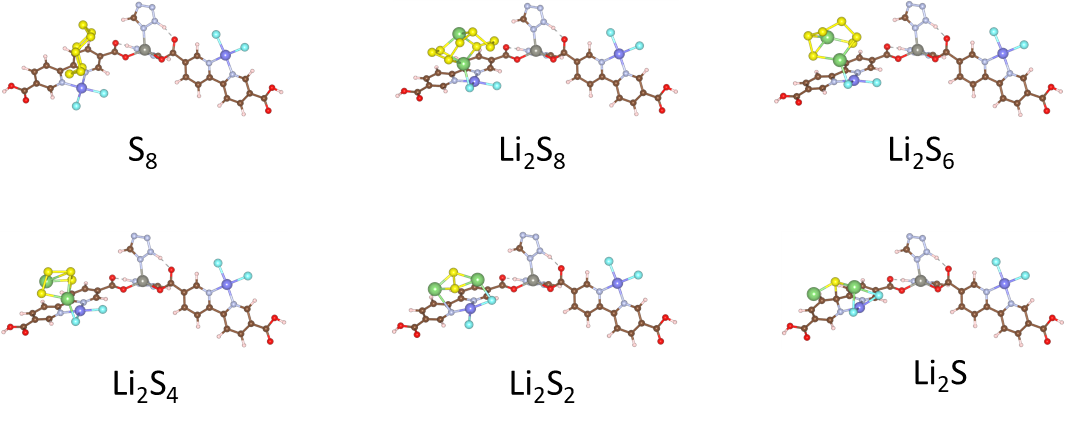


**Fig. S20** Optimal structural model for adsorption of polysulfides by Ni-AHF-DPDC


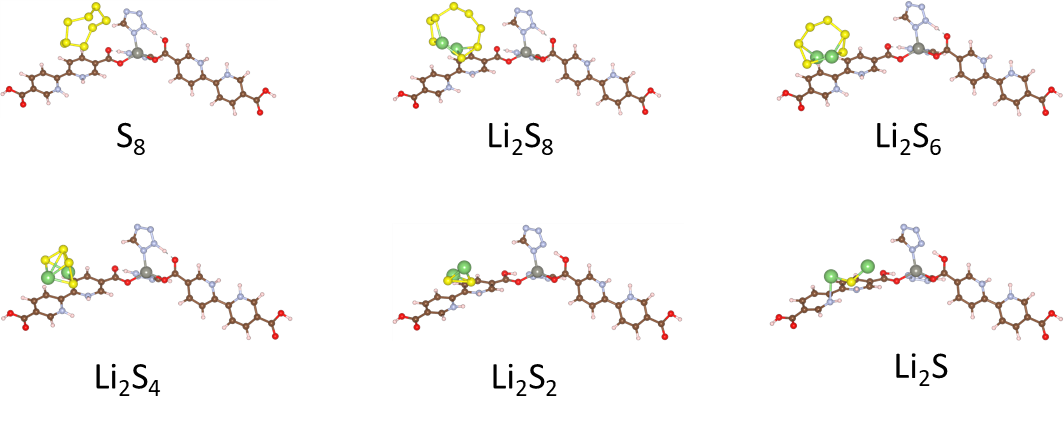


**Fig. S21** Optimal structural model for adsorption of polysulfides by AHF-DPDC


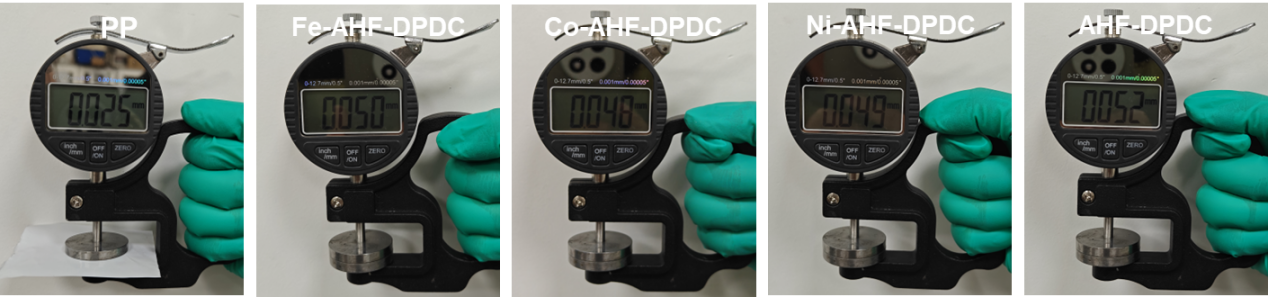


**Fig. S22** Results of coating thickness analysis for the modified separator


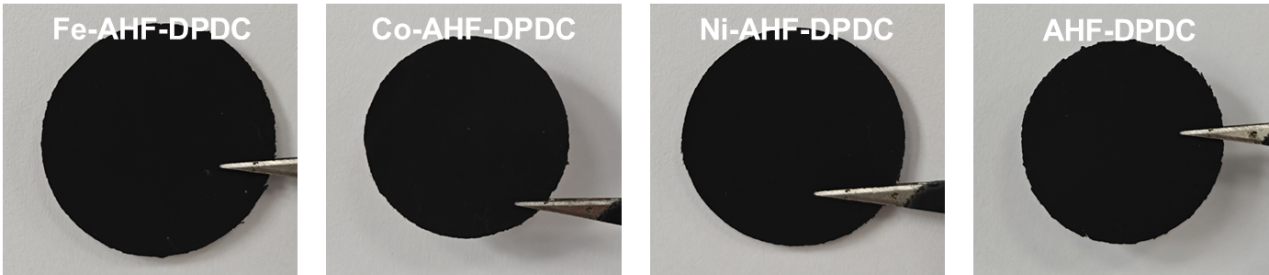


**Fig. S23** Surface optical photographs of the modified separator


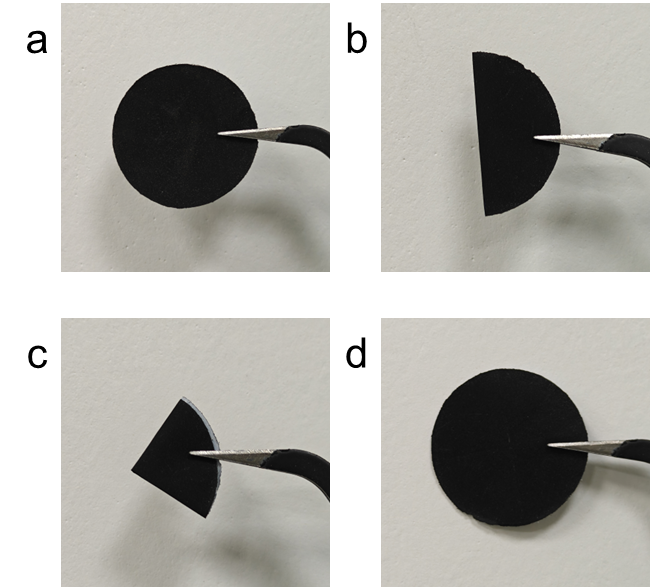


**Fig. S24** Digital images of Fe-AHF-DPDC modified separator (**a**), single (**b**) and double folded (**c**), and recovered (**d**)


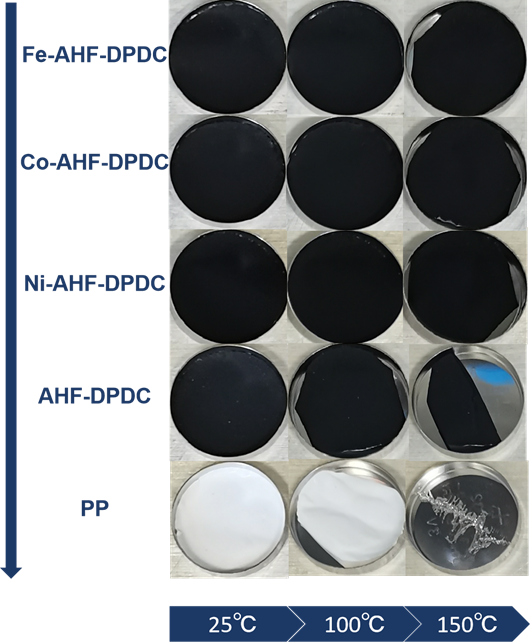


**Fig. S25** The physical heating tests of Fe-AHF-DPDC modified separator, Co-AHF-DPDC, Ni-AHF-DPDC, Ni-DPDC-AHF modified separator and PP separators at 25, 100 and 150 ℃


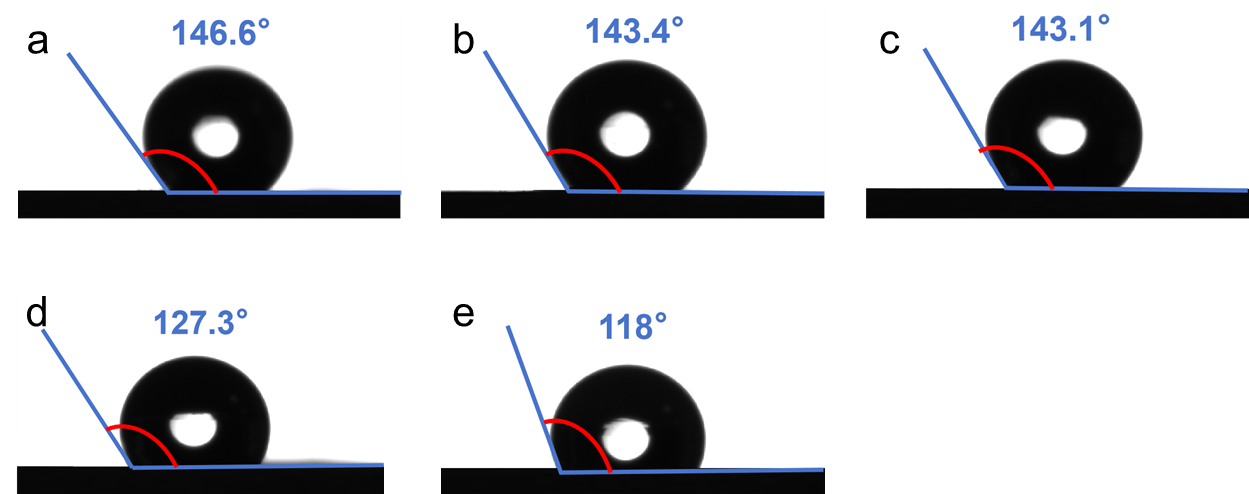


**Fig. S26** Water wetting test of Fe-AHF-DPDC (**a**), Co-AHF-DPDC (**b**), Ni-AHF-DPDC (**c**), AHF-DPDC modified separator (**d**) and PP separator (**e**)


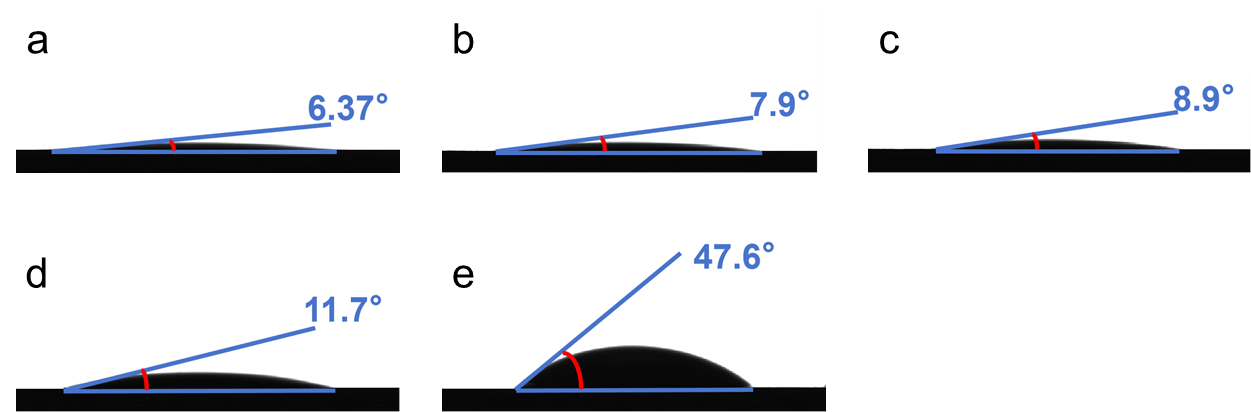


**Fig. S27** Electrolyte wetting test of Fe-AHF-DPDC (**a**), Co-AHF-DPDC (**b**), Ni-AHF-DPDC (**c**), AHF-DPDC modified separator (**d**) and PP separator (**e**)


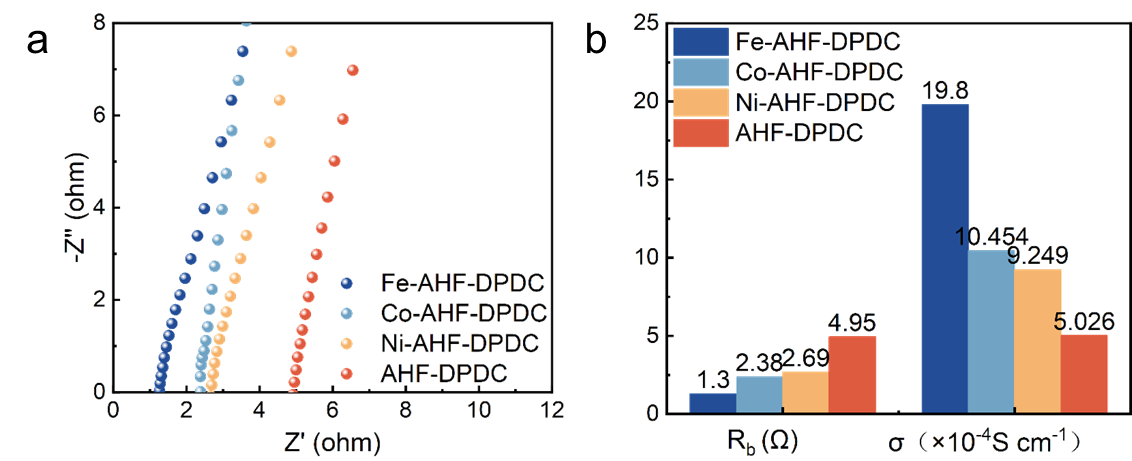


**Fig. S28** Ionic conductivities and resistance of Fe-AHF-DPDC, Co-AHF-DPDC, Ni-AHF-DPDC, and AHF-DPDC cells


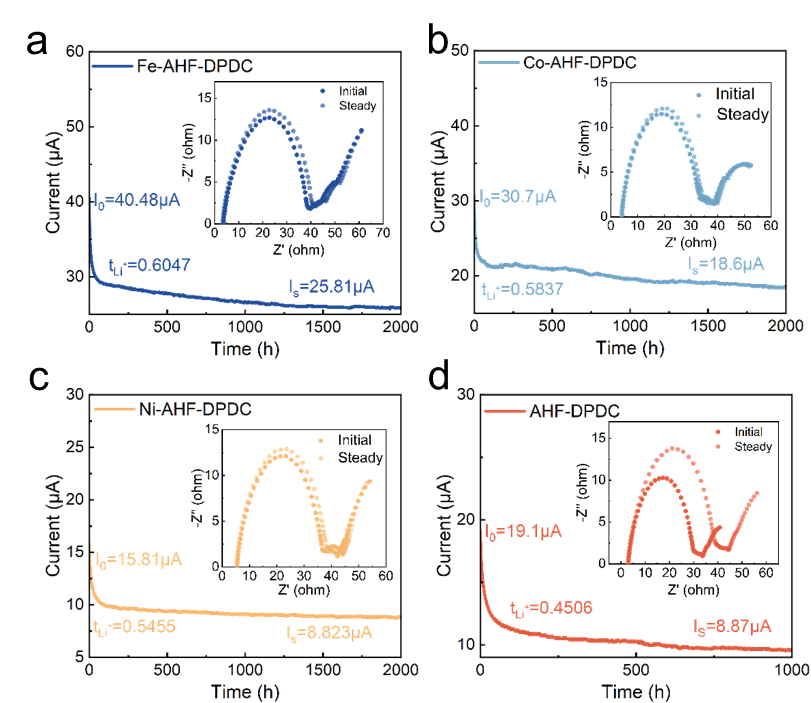


**Fig. S29** The time-dependence response of potentiostatic DC polarization for symmetrical Li/separator/Li symmetric cells with Fe-AHF-DPDC (**a**), Co-AHF-DPDC (**b**), Ni-AHF-DPDC (**c**) and AHF-DPDC (**d**) cells, respectively, the insets are the Nyquist plots of tested cells before/after polarization at room temperature


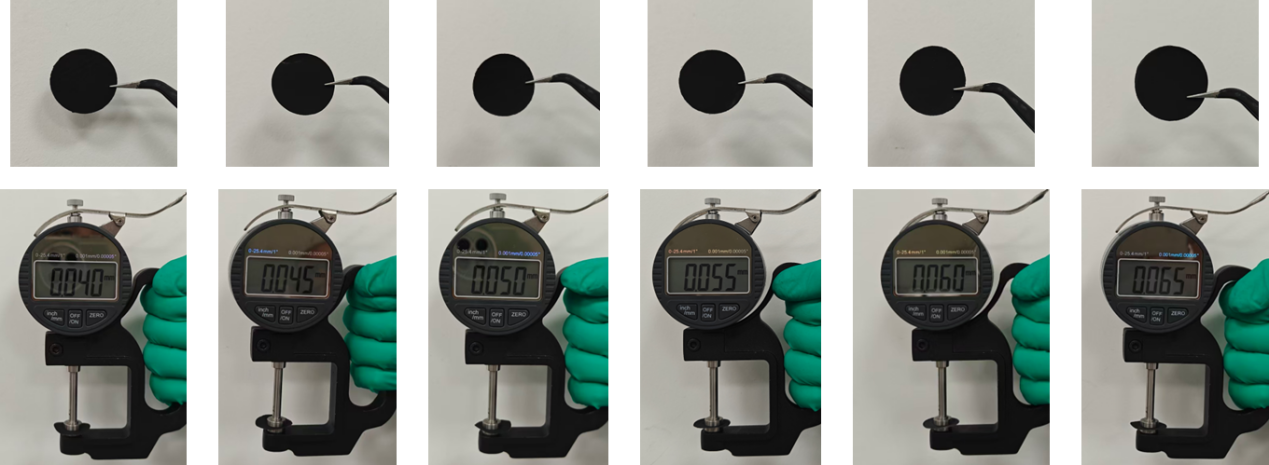


**Fig. S30** Surface optical photographs and coating thickness analysis of Fe-AHF-DPDC modified separators fabricated via doctor blade coating with varying thicknesses


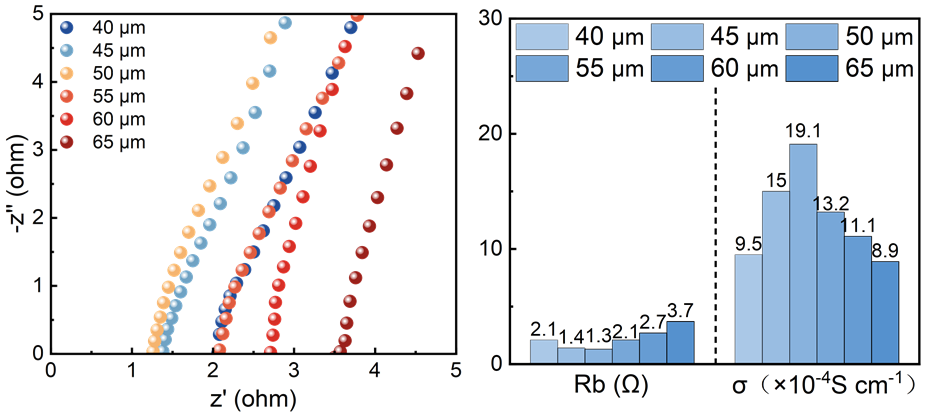


**Fig. S31** EIS plots and ionic conductivity of Fe-AHF-DPDC-modified separators of different thicknesses


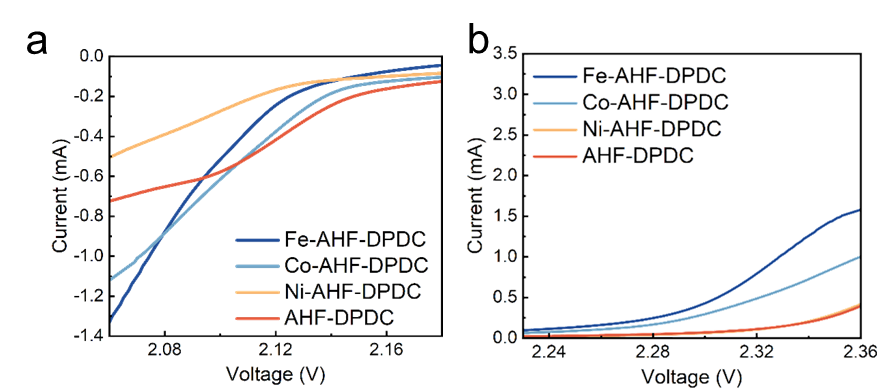


**Fig. S32** The LSV of Peak Ⅱ (**a**) and Peak Ⅲ (**b**) at different voltage windows


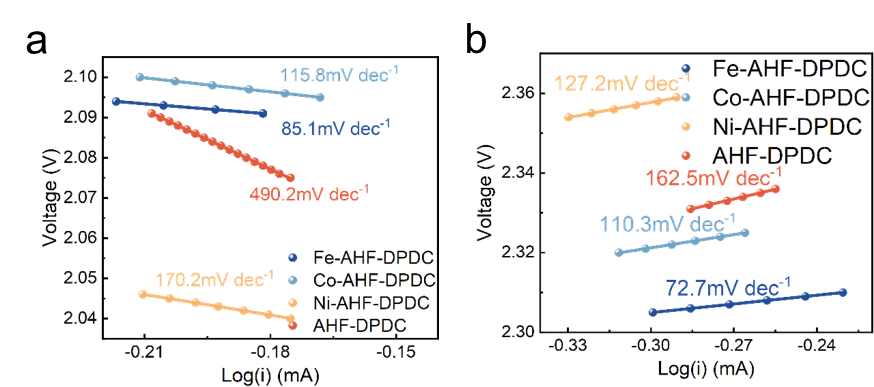


**Fig. S33** The inset is the corresponding Tafel diagram corresponding to Peak Ⅱ (a), and Peak Ⅲ(b). A smaller Tafel slope implies a faster kinetic process, indicating that the active substance could reach the desired current at a lower overpotential


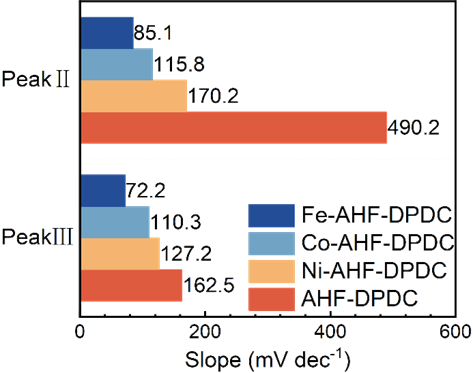


**Fig. S34** Columnar comparison of Tafel slopes with different diaphragms


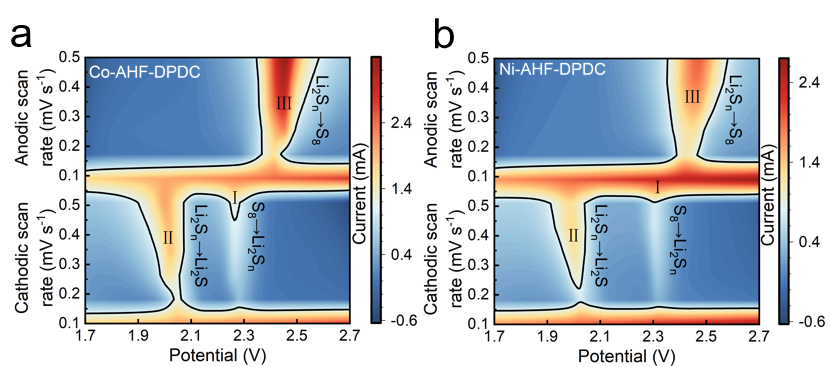


**Fig. S35** CV curves of Co-AHF-DPDC (**a**) and Ni-AHF-DPDC (**b**) cells at different scan rates


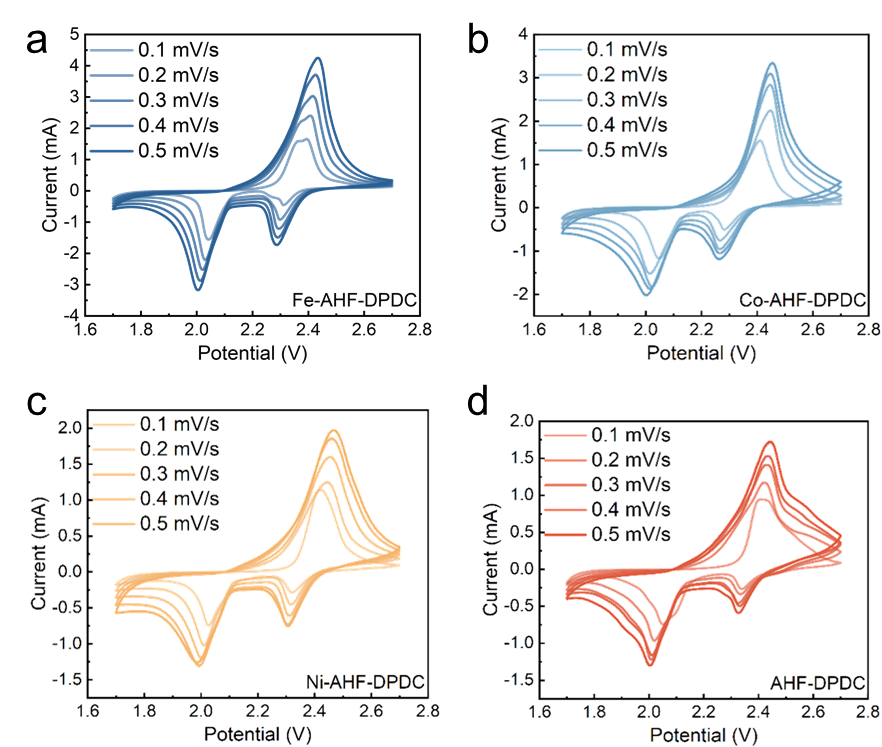


**Fig. S36** CV curves at different scan rates. Fe-AHF-DPDC (**a**), Co-AHF-DPDC (**b**), Ni-AHF-DPDC (**c**), and AHF-DPDC(**d**) cells


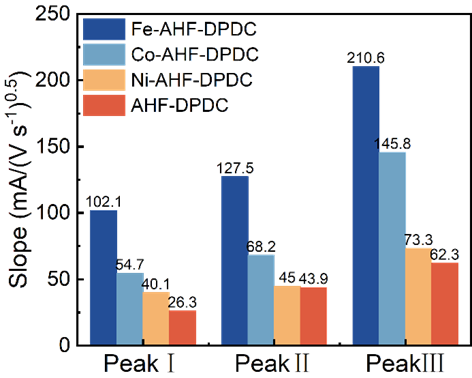


**Fig. S37** A columnar comparison of the slope of the line fitted by the three peaks with different separators


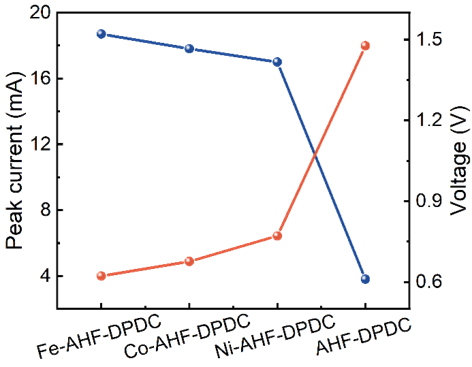


**Fig. S38** Line plot of peak current and overpotential derived from cyclic voltammetry curves of symmetrical cells of Fe-AHF-DPDC, Co-AHF-DPDC, Ni-AHF-DPDC and AHF-DPDC


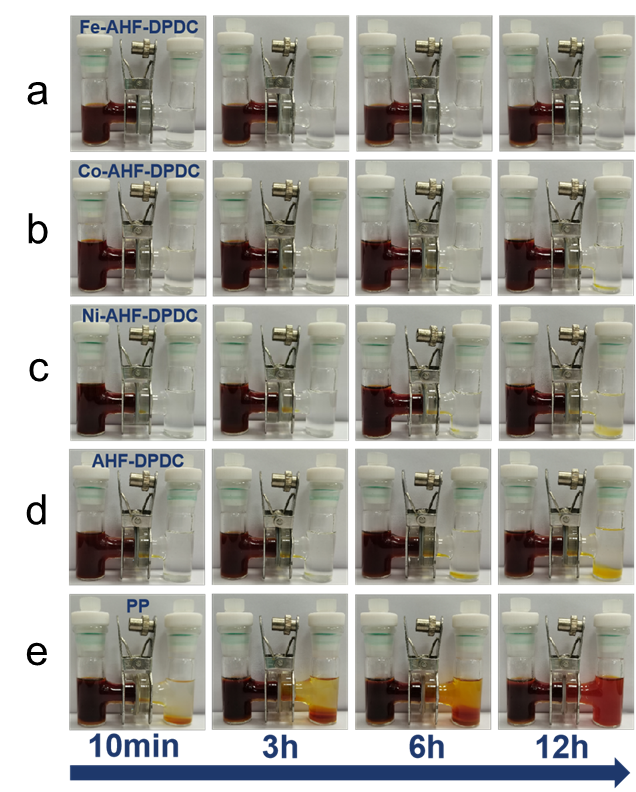


**Fig. S39** Permeation experiment with the H-type permeation device for the Fe-AHF-DPDC (**a**), Co-AHF-DPDC (**b**), Ni-AHF-DPDC (**c**), AHF-DPDC(**d**) modified separators and PP (**e**) separators


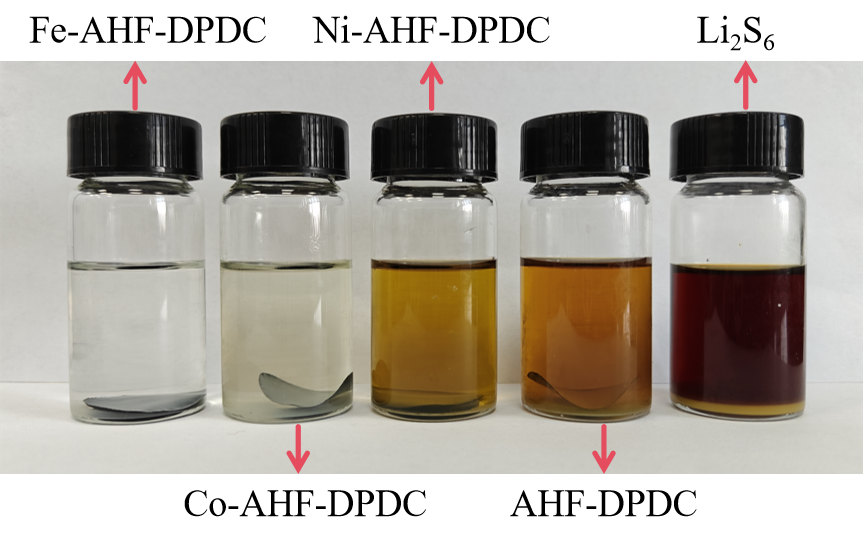


**Fig. S40** Li_2_S_6_ solution after 24 h of adsorption by Fe-AHF-DPDC, Co-AHF-DPDC, Ni-AHF-DPDC, and AHF-DPDC


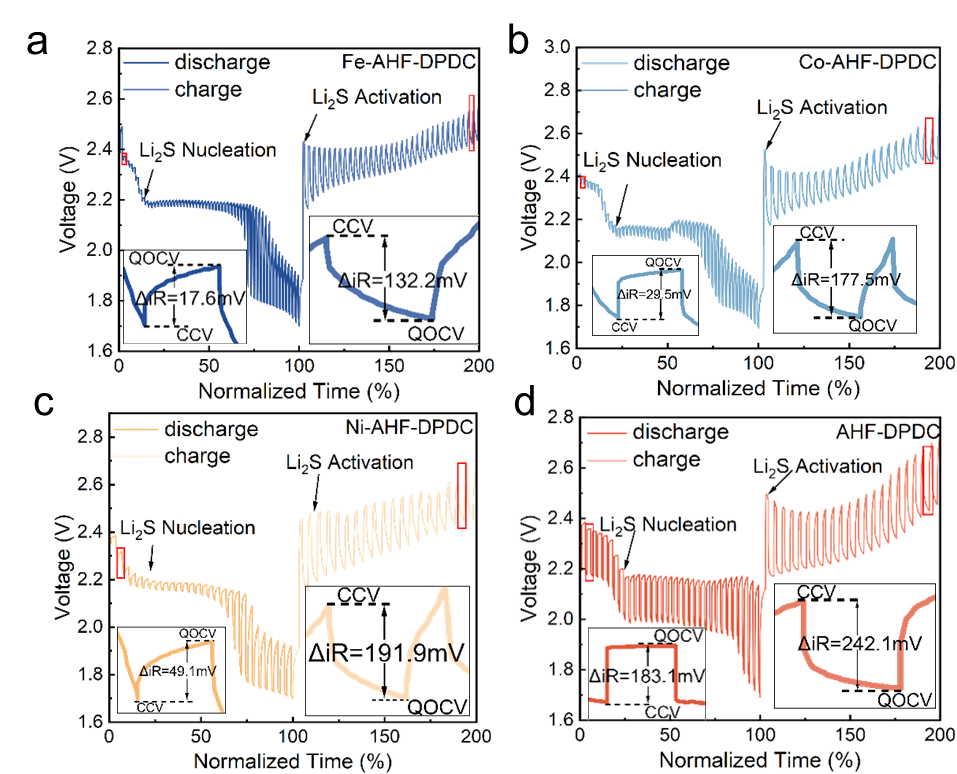


**Fig. S41** The GITT plots of Li-S batteries for Co-AHF-DPDC (**a**) and Ni-AHF-DPDC (**b**) cells in the second discharge/charge process under the current density of 0.1 C


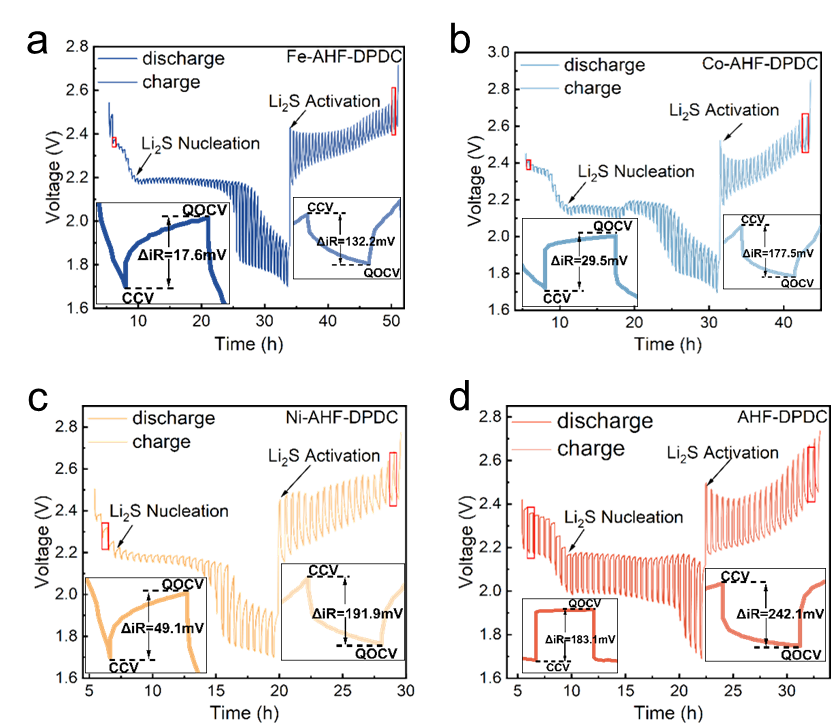


**Fig. S42** The GITT plots of Li-S batteries for Fe-AHF-DPDC (**a**)、Co-AHF-DPDC (**b**)、Ni-AHF-DPDC (**c**) and AHF-DPDC (**d**) cells in the second discharge/charge process under the current density of 0.1 C


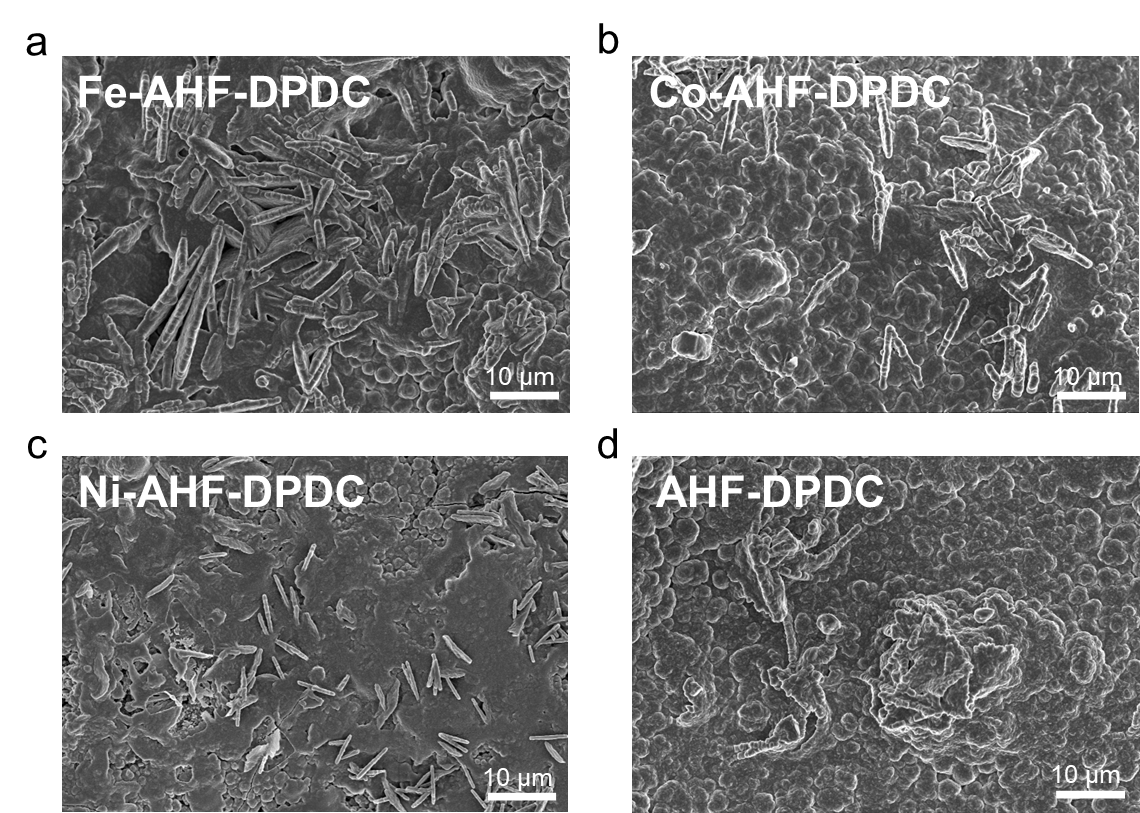


**Fig. S43** SEM images of Fe-AHF-DPDC (**a**)、Co-AHF-DPDC (**b**)、Ni-AHF-DPDC (**c**) and AHF-DPDC(**d**) after Li_2_S nucleation tests


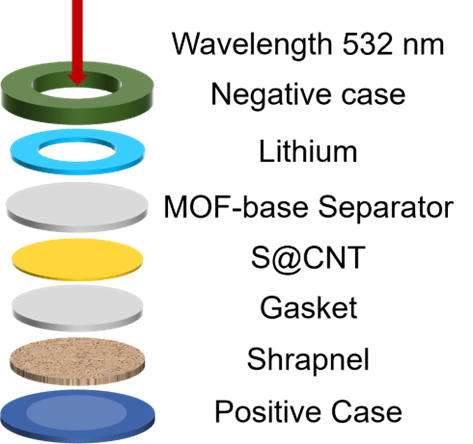


**Fig. S44** The cell configuration with a sealed glass window for in situ Raman analysis


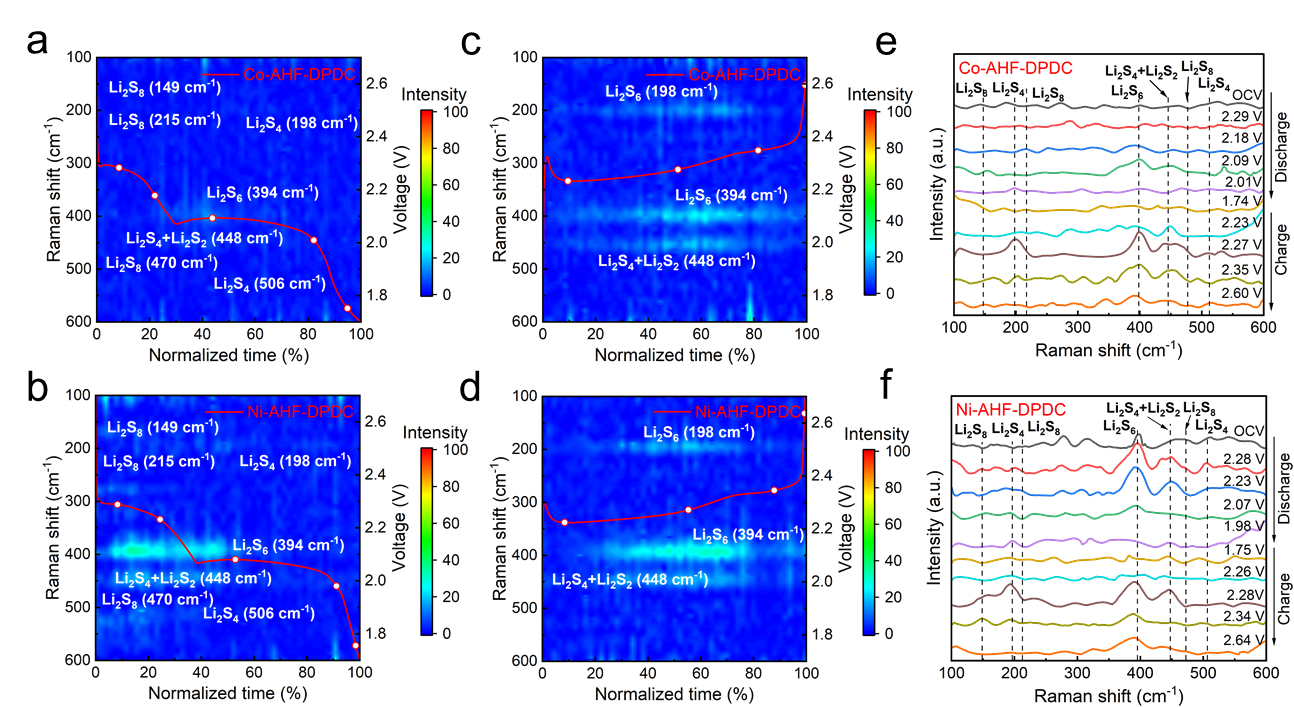


**Fig. S45** Time-resolved Raman spectra at 0.1 C for the discharge and charge process for Co-AHF-DPDC (**a, c, e**) and Ni-AHF-DPDC (**b, d, f**) cells


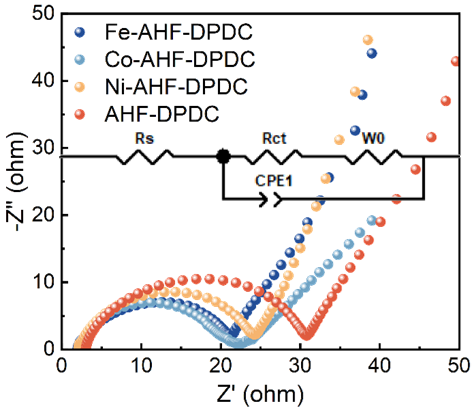


**Fig. S46** Impedance plots of Fe-AHF-DPDC, Co-AHF-DPDC, Ni-AHF-DPDC, and AHF-DPDC assembled lithium-sulfur batteries


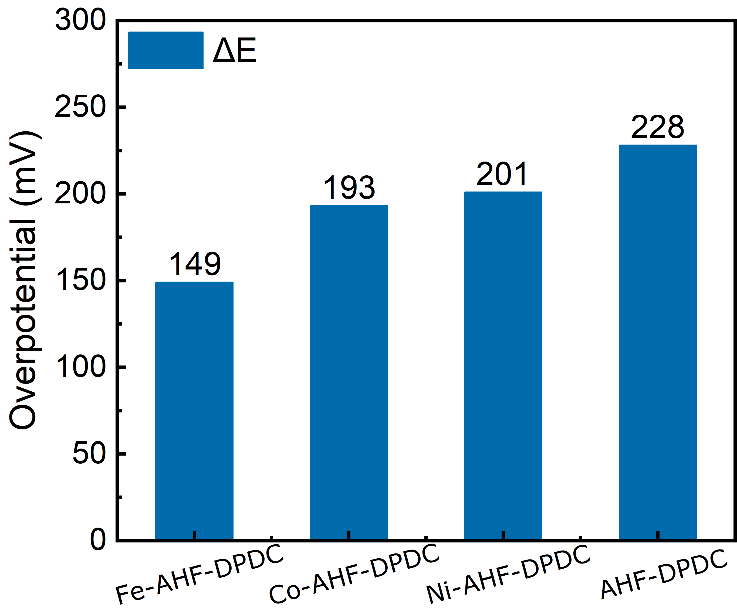


**Fig. S47** The overpotential capacities for Fe-AHF-DPDC, Co-AHF-DPDC, Ni-AHF-DPDC and AHF-DPDC cells at 0.1 C


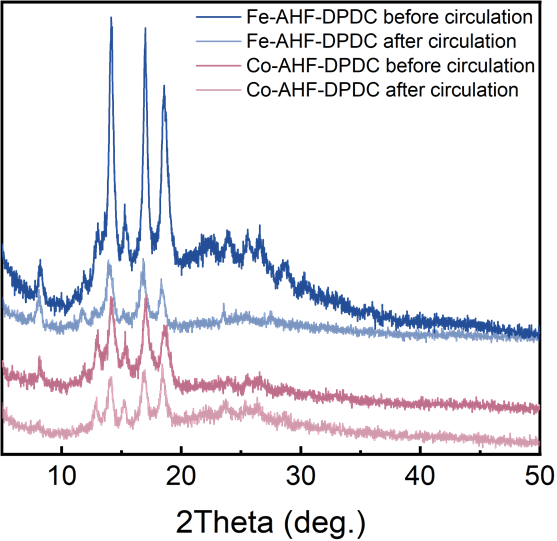


**Fig. S48** XRD of Fe-AHF-DPDC modified separator pre- and post-50 cycles (0.1 C)


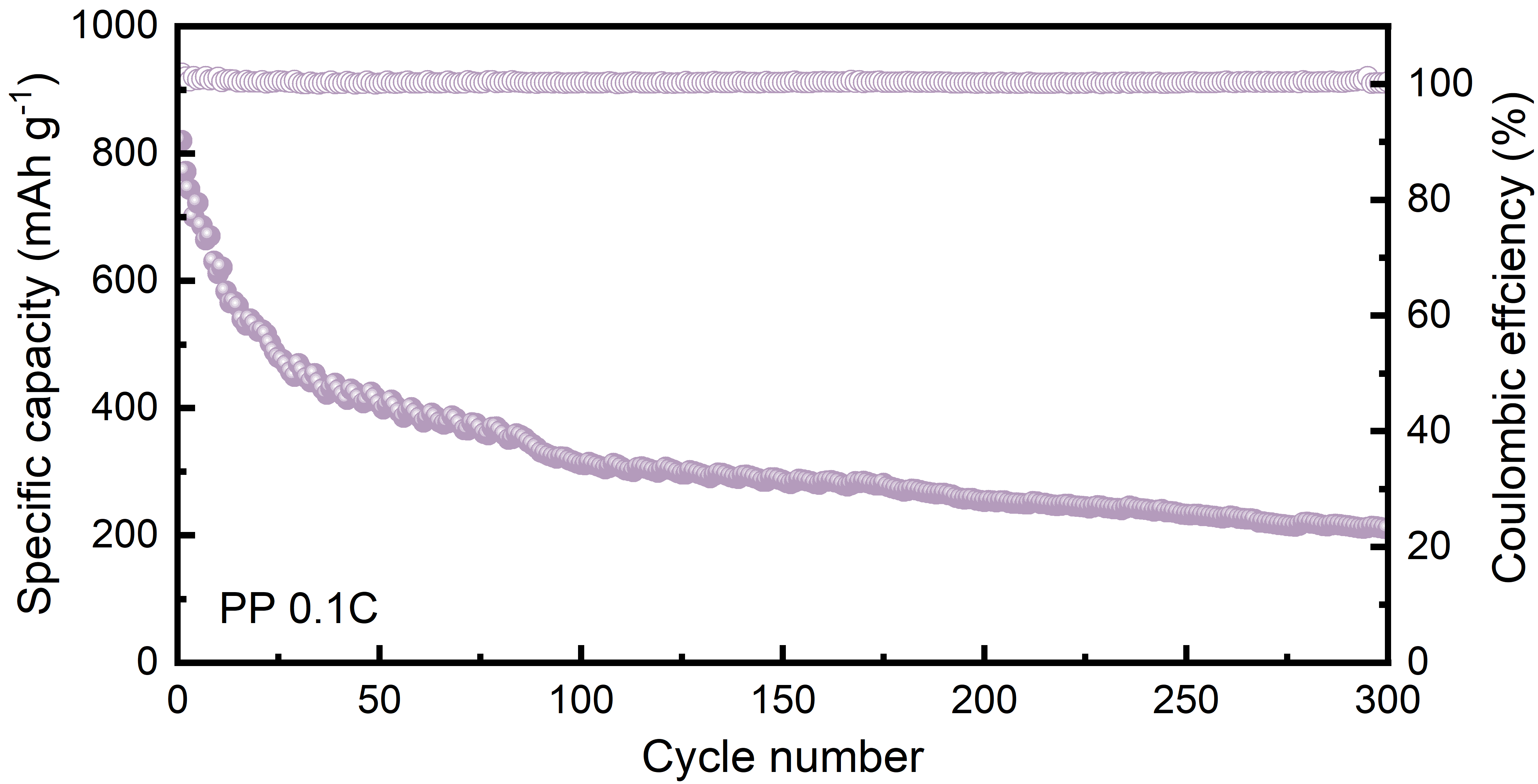


**Fig. S49** Cycle diagram of lithium-sulfur batteries at 0.1 C using PP separators


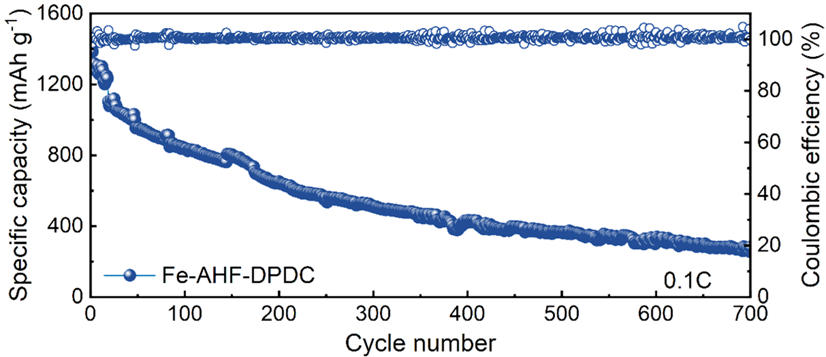


**Fig. S50** Diagram of a 700-turn cycle of a lithium-sulfur battery using a Fe-AHF-DPDC separator at 0.1 C


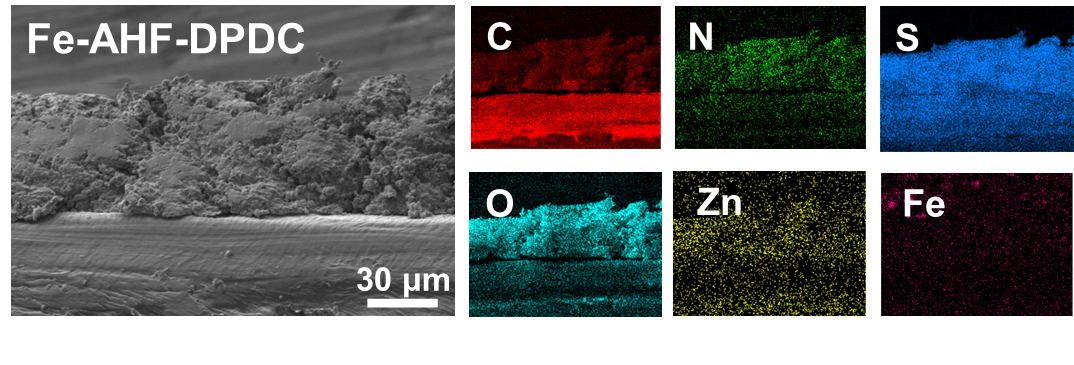


**Fig. S51** Cross-sectional view of the separators. SEM images of Fe-AHF-DPDC modified separators with corresponding C, N, S, O, Zn and Fe element mappings after Li-S cell cycling


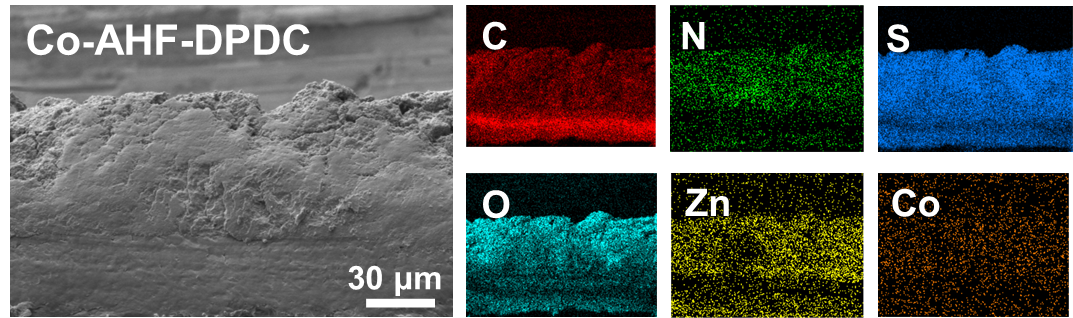


**Fig. S52** Cross-sectional view of the separators. SEM images of Co-AHF-DPDC modified separators with corresponding C, N, S, O, Zn and Co element mappings after Li-S cell cycling


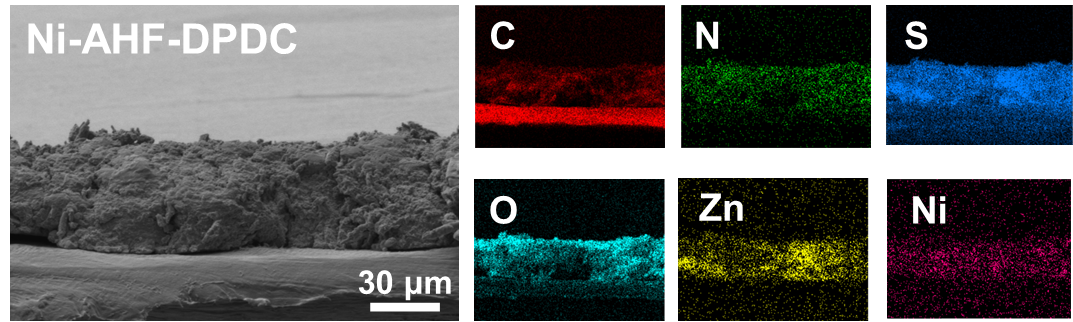


**Fig. S53** Cross-sectional view of the separators. SEM images of Ni-AHF-DPDC modified separators with corresponding C, N, S, O, Zn and Ni element mappings after Li-S cell cycling


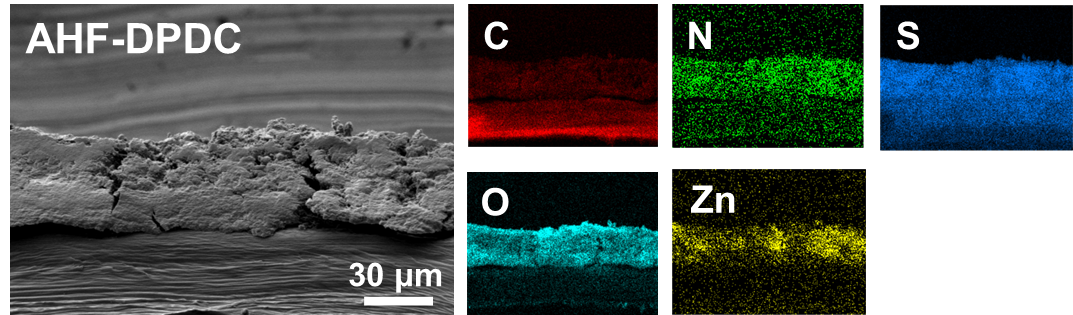


**Fig. S54** Cross-sectional view of the separators. SEM images of AHF-DPDC modified separators (d) with corresponding C, N, S, O and Zn element mappings after Li-S cell cycling


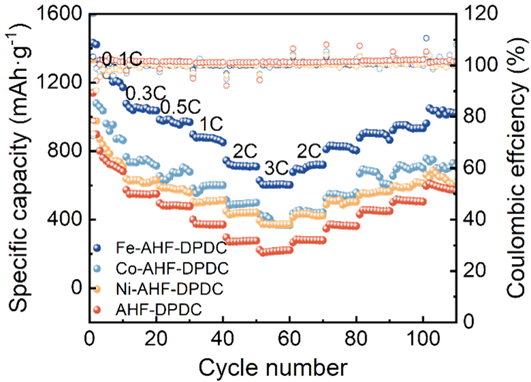


**Fig. S55** Rate performance of lithium-sulfur batteries of Fe-AHF-DPDC, Co-AHF-DPDC, Ni-AHF-DPDC, and AHF-DPDC at different current densities


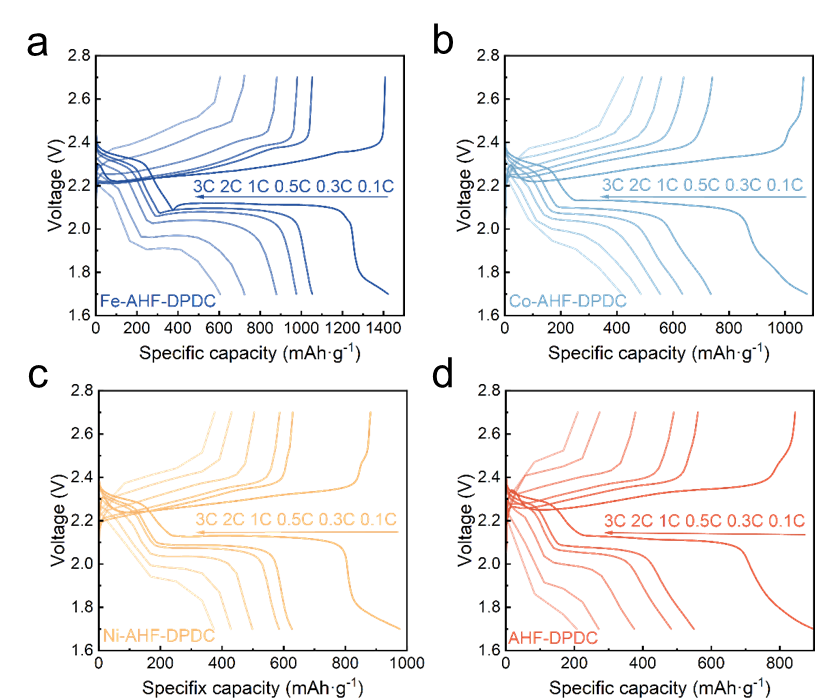


**Fig. S56** Discharge-charge profiles of Fe-AHF-DPDC (**a**), Co-AHF-DPDC (**b**), Ni-AHF-DPDC (**c**) and AHF-DPDC (**d**) cells at the rate current from 0.1 to 3 C


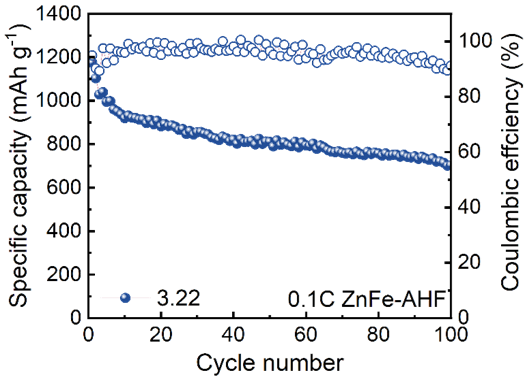


**Fig. S57** Cycling performance under high sulfur loading


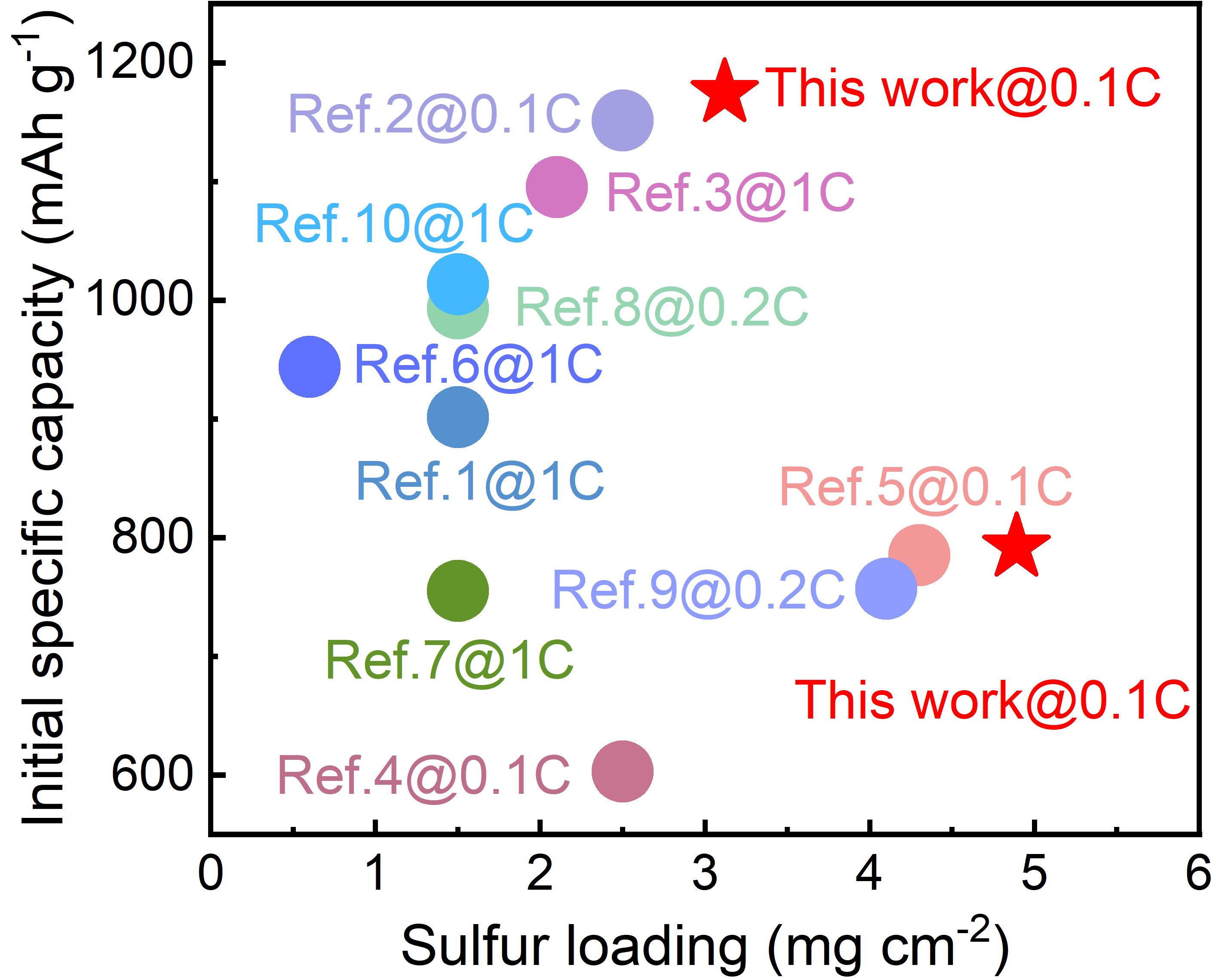


**Fig. S58** A literature comparison chart of Fe-AHF-DPDC with other separators [S7-S16].


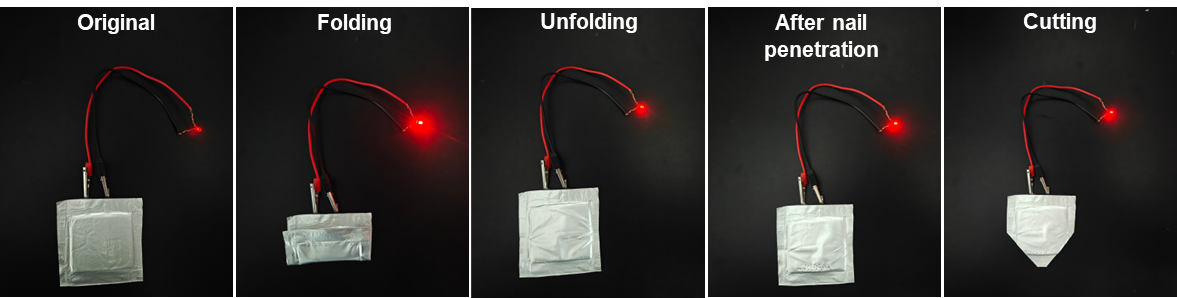


**Fig. S59** Photo of the lithium sulfur pouch battery with Fe-AHF-DPDC separators lighted up the LEDs

**Table S1** ICP Analysis of Zn and Co in AHF-BPDC Before and After Ion Exchange Reaction

| **Sample Name** | **Zn%** | **Co%** |
| --- | --- | --- |
| AHF-BPDC | 16.2% | 0% |
| AHF-BPDC after ion exchange reaction | 16.4% | 0.11% |

**Table S2** Comparison of Rate Performance Among Fe-AHF-DPDC, Co-AHF-DPDC, Ni-AHF-DPDC, and AHF-DPDC Modified Separators

| **Initial specific capacity**  **at various rates (mAh g^-1^)** | **0.1C** | **0.3C** | **0.5C** | **1C** | **2C** | **3C** |
| --- | --- | --- | --- | --- | --- | --- |
| Fe-AHF-DPDC | 1431.9 | 1076.8 | 983.9 | 898.1 | 745.7 | 628.9 |
| Co-AHF-DPDC | 1604.8 | 766.3 | 654.3 | 581.6 | 514.5 | 431 |
| Ni-AHF-DPDC | 1334.8 | 638.8 | 595.1 | 504.8 | 449.8 | 393.9 |
| AHF-DPDC | 1140.3 | 574.1 | 496.6 | 400.9 | 296.5 | 223.6 |

**Table S3** Battery performance comparison: functional organic polymer MOF modified separator for lithium-sulfur batteries

| **Separators** | **Sulfur loading**  **(mg cm^-2^)** | **Initial capacity**  **(mAh g^-1^)** | **Cycle number** | **Rate (C)** | **Pubish year** | **Refs.** |
| --- | --- | --- | --- | --- | --- | --- |
| Fe-AHF-DPDC | 1 | 1400.7 | 700 | 0.1 | -- | This work |
| MoB | 1.2 | 847 | 500 | 2.0 | 2025 | [S17] |
| MIP-202@2320 | -- | 855 | 600 | 0.5 | 2024 | [S18] |
| D-ZIF-L | -- | 897.3 | 500 | 1.0 | 2024 | [S19] |
| HPC-MOF-M | 1.5 | 1013.3 | 500 | 1.0 | 2024 | [S16] |
| Fe-ZIF-8/PP | 2.5 | 603 | 100 | 0.1 | 2023 | [S10] |
| NCMP | -- | 794 | 500 | 1.0 | 2023 | [S20] |
| Ni@C-CNT | 1.3 | 903 | 600 | 1.0 | 2023 | [S8] |
| np-ANF | 3.6 | 1119 | 200 | 0.1 | 2022 | [S21] |
| PMIA/Celgard | 0.6 | 944 | 600 | 1.0 | 2020 | [S12] |
| TPE@PP | 1.5 | 755 | 600 | 1.0 | 2020 | [S13] |

|  |
| --- |

**Supplementary References**

[S1] G. Kresse, J. Hafner, *Ab initio*molecular dynamics for liquid metals. Phys. Rev. B **47**(1), 558–561 (1993). <https://doi.org/10.1103/physrevb.47.558>

[S2] G. Kresse, J. Hafner, *Ab initio*molecular-dynamics simulation of the liquid-metal–amorphous-semiconductor transition in germanium. Phys. Rev. B **49**(20), 14251–14269 (1994). <https://doi.org/10.1103/physrevb.49.14251>

[S3] J.P. Perdew, K. Burke, M. Ernzerhof, Generalized gradient approximation made simple. Phys. Rev. Lett. **77**(18), 3865–3868 (1996). <https://doi.org/10.1103/physrevlett.77.3865>

[S4] G. Kresse, D. Joubert, From ultrasoft pseudopotentials to the projector augmented-wave method. Phys. Rev. B **59**(3), 1758–1775 (1999). <https://doi.org/10.1103/physrevb.59.1758>

[S5] S. Grimme, J. Antony, S. Ehrlich, H. Krieg, A consistent and accurate *ab initio* parametrization of density functional dispersion correction (DFT-D) for the 94 elements H-Pu. J. Chem. Phys. **132**(15), 154104 (2010). <https://doi.org/10.1063/1.3382344>

[S6] P. Yang, J. Qiang, J. Chen, Z. Zhang, M. Xu et al., A versatile metal-organic-framework pillared interlayer design for high-capacity and long-life lithium-sulfur batteries. Angew. Chem. Int. Ed. **64**(2), e202414770 (2025). <https://doi.org/10.1002/anie.202414770>

[S7] K. Wang, Y. Wang, J. Wang, H. Wang, C. Ding et al., Unveiling the curvature-dependent electrocatalytic kinetics for sulfur redox reaction in Li-S chemistry. Adv. Funct. Mater. **35**(26), 2422689 (2025). <https://doi.org/10.1002/adfm.202422689>

[S8] X. Ao, Y. Kong, S. Zhao, Z. Chen, Y. Li et al., Metal-N coordination in lithium-sulfur batteries: inhibiting catalyst passivation. Angew. Chem. Int. Ed. **64**(3), e202415036 (2025). <https://doi.org/10.1002/anie.202415036>

[S9] S. Guo, Y. Xiao, A. Cherevan, D. Eder, L. Xu et al., Catalytic multivariable metal-organic frameworks for lithium-sulfur batteries. Mater. Today **65**, 37–46 (2023). <https://doi.org/10.1016/j.mattod.2023.03.019>

[S10] R. Razaq, M.M.U. Din, D.R. Småbråten, V. Eyupoglu, S. Janakiram et al., Synergistic effect of bimetallic MOF modified separator for long cycle life lithium-sulfur batteries. Adv. Energy Mater. **14**(3), 2302897 (2024). <https://doi.org/10.1002/aenm.202302897>

[S11] H. Li, W. Zheng, H. Wu, Y. Fang, L. Li et al., Ultra-dispersed α-MoC_1–_*_x_* embedded in a plum-like N-doped carbon framework as a synergistic adsorption–electrocatalysis interlayer for high-performance Li–S batteries. Small **20**(10), 2306140 (2024). https://doi.org/10.1002/smll.202306140

[S12] C.-C. Sun, Y.-Z. Song, Y. Yan, J.-J. Yuan, Z. Huang et al., Integrating flexible PMIA separator and electrode for dealing with multi-aspect issues in Li–S batteries. Electrochim. Acta **359**, 136987 (2020). <https://doi.org/10.1016/j.electacta.2020.136987>

[S13] Z. Zhao, Y. Pan, H. Chen, X. Wang, Y. Huang et al., A lithium-affinitive covalent organic polymer network functionalized separator for dendrite-free and high-durability lithium–sulfur batteries under harsh conditions. Adv. Funct. Mater. **34**(37), 2402182 (2024). <https://doi.org/10.1002/adfm.202402182>

[S14] X. Ren, Q. Wang, Y. Pu, Q. Sun, W. Sun et al., Synergizing spatial confinement and dual-metal catalysis to boost sulfur kinetics in lithium–sulfur batteries. Adv. Mater. **35**(44), 2304120 (2023). <https://doi.org/10.1002/adma.202304120>

[S15] Q. Lv, Y. Sun, B. Li, C. Li, Q. Zhang et al., Metal–organic frameworks with axial cobalt–oxygen coordination modulate polysulfide redox for lithium–sulfur batteries. Adv. Energy Mater. **15**(5), 2403223 (2025). <https://doi.org/10.1002/aenm.202403223>

[S16] L. Xie, Y. Xiao, Q. Zeng, Y. Wang, J. Weng et al., Balanced mass transfer and active sites density in hierarchical porous catalytic metal–organic framework for enhancing redox reaction in lithium–sulfur batteries. ACS Nano **18**(20), 12820–12829 (2024). <https://doi.org/10.1021/acsnano.3c13087>

[S17] J. Pu, S. Fan, Z. Shen, J. Yin, Y. Tan et al., F-free fabrication novel 2D Mo-based MBene catalyst for advanced lithium–sulfur batteries. Adv. Funct. Mater. **35**(25), 2424215 (2025). <https://doi.org/10.1002/adfm.202424215>

[S18] L. Zhou, H. Pan, G. Yin, Y. Xiang, P. Tan et al., Tailoring the function of battery separators *via* the design of MOF coatings. Adv. Funct. Mater. **34**(23), 2314246 (2024). <https://doi.org/10.1002/adfm.202314246>

[S19] J. Wang, X. Zhang, X. Wang, J. Liu, S. Li et al., Activation of MOF catalysts with low steric hindrance *via* undercoordination chemistry for efficient polysulfide conversion in lithium–sulfur battery. Adv. Energy Mater. **14**(37), 2402072 (2024). <https://doi.org/10.1002/aenm.202402072>

[S20] X. Leng, J. Zeng, M. Yang, C. Li, S.V.P. Vattikuti et al., Bimetallic Ni–Co MOF@PAN modified electrospun separator enhances high-performance lithium-sulfur batteries. J. Energy Chem. **82**, 484–496 (2023). <https://doi.org/10.1016/j.jechem.2023.03.017>

[S21] M. Wang, A.E. Emre, J.-Y. Kim, Y. Huang, L. Liu et al., Multifactorial engineering of biomimetic membranes for batteries with multiple high-performance parameters. Nat. Commun. **13**, 278 (2022). <https://doi.org/10.1038/s41467-021-27861-w>
